# Using the isotope effect to probe an aggregation induced emission mechanism: theoretical prediction and experimental validation†
†Electronic supplementary information (ESI) available: Computational details, normal mode analysis, synthesis and characterization. See DOI: 10.1039/c6sc00839a


**DOI:** 10.1039/c6sc00839a

**Published:** 2016-05-11

**Authors:** Tian Zhang, Qian Peng, Changyun Quan, Han Nie, Yingli Niu, Yujun Xie, Zujin Zhao, Ben Zhong Tang, Zhigang Shuai

**Affiliations:** a Key Laboratory of Organic OptoElectronics and Molecular Engineering , Department of Chemistry , Tsinghua University , Beijing , 100084 , China . Email: zgshuai@tsinghua.edu.cn; b Key Laboratory of Organic Solids , Beijing National Laboratory for Molecular Science (BNLMS) , Institute of Chemistry , Chinese Academy of Sciences , Beijing , 100190 , China . Email: qpeng@iccas.ac.cn; c State Key Laboratory of Luminescent Materials and Devices , South China University of Technology , Guangzhou , 510640 , China; d National Center for Nanoscience and Technology , Chinese Academy of Sciences , Beijing , 100190 , China; e Department of Chemistry , Wuhan University , Wuhan , 430072 , China; f Collaborative Innovation Center of Chemistry for Energy Materials , Xiamen University , Xiamen , 351005 , China

## Abstract

Aggregation-induced emission (AIE) has become a hot topic for a variety of potential applications, but the understanding of its working mechanism is still under scrutiny. Herein, we proposed the use of the isotope effect (IE) to identify the AIE mechanism: under the restriction of an internal motion mechanism, the IE is pronouncedly different in excited-state decay rates when contrasting AIE luminogens (AIEgens) and non-AIEgens in theoretical calculations. For the complete deuteration of AIEgens, the IE of nonradiative decay rate in solution (<–10%) is much weaker than that (–65% to –95%) in aggregate, because the former stems from the overall results of competitive vibronic coupling and the severe mixing of low-frequency modes while the latter mainly comes from the vibronic coupling only. The experimental results confirm the isotopic “jump” behaviors in AIEgens well. However, non-AIEgens exhibit equivalent IEs (–40% to –90%) in both solution and solid phases. Further partial deuteration schemes for the 6-ring AIE analogues show positional dependence.

## Abstract 2

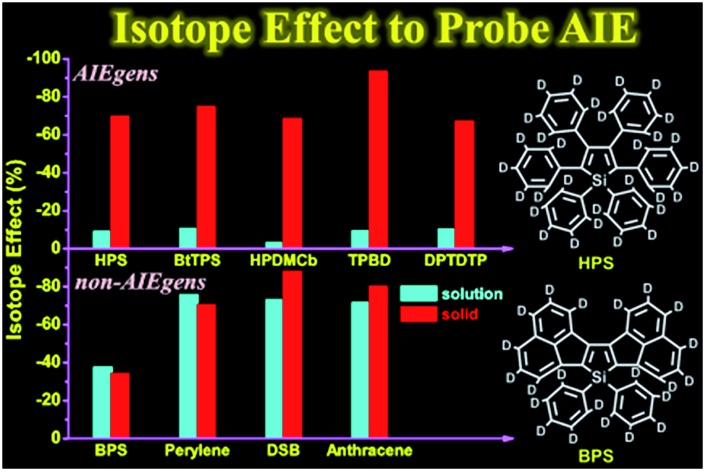
The isotope effect (IE) on non-radiative decay is proposed theoretically and verified with experiments. For AIEgens, the IE is much stronger in aggregate than in solution. For non-AIEgens, both phases exhibit appreciable and similar IEs.

## Introduction

Molecular aggregation in general tends to reduce luminescence efficiency: intermolecular charge transfer or energy transfer to quenching sites, or the formation of a dipole-forbidden lowest excited state.^1^ However, a series of aggregation induced emissive luminogens (AIEgens) are found to be poorly luminescent in dilute solution but highly emissive in an aggregate or crystalline state, demonstrating potential application in organic solid-state lighting and cell imaging.^2^ Despite the varieties of experimental efforts to clarify the AIE mechanism, there are still controversies regarding the microscopic origin of AIE: restriction of intramolecular motion (RIM),3*a* J-aggregation formation,3b,c hydrogen-bond induced excimer-emission,3*d* intramolecular planarization,3*e* vibration induced emission,3*f* and so on. To better understand AIE, here we propose to use the isotope effect (IE) to probe the influence of molecular aggregation on the excited-state non-radiative decay from the lowest excited singlet state (S_1_) to the ground state (S_0_) through comparative computational studies for AIEgens with RIM mechanisms and conventional luminogens.

Deuteration has been widely applied to probe excited-state decay processes.^4^ For conventional fluorophores, it is known that deuteration always causes a decrease in the nonradiative decay rate while hardly changing the radiative decay rate, leading to an increase in luminescence efficiency. This point is easily understood from the internal conversion rate under the displaced harmonic oscillator model based on the Fermi golden rule, where the *k*_ic_ is exponentially proportional to –*S*_*j*_, the Huang–Rhys factor of the *j*-th normal mode, which is determined by the normal mode reorganization energy *λ*_*j*_ = *ħS*_*j*_*ω*_*j*_.^5^ According to the four-point method in the potential energy surface, the total reorganization energy summated over all the normal modes is also defined as *λ*_g(e)_ = *E*_g(e)_(S_1(0)_-geometry) – *E*_g(e)_(S_0(1)_-geometry) in the ground (excited) state. Thus, it is independent of isotopic substitution since the equilibrium geometry and electronic-state energy are the same for different isotopomers.^6^ The lower frequency *ω*_*j*_ induced by the deuteration of the modes implies the increase of the Huang–Rhys factor *S*_*j*_, which reduces the *k*_ic_ with the displaced approximation.

**Chart 1:**
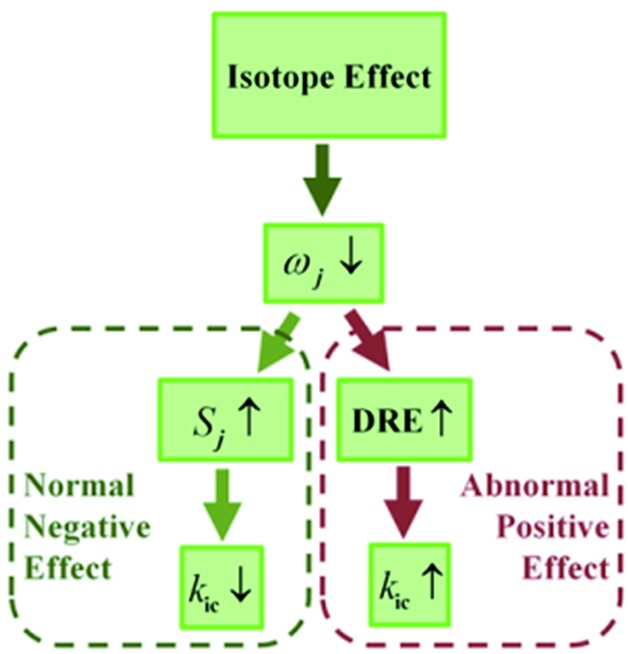
Representation of isotope effect on *k*_ic_.

For the flexible AIEgens, mixing between the low-frequency normal modes is significant and the *k*_ic_ cannot be correctly described under the displaced harmonic oscillator model. Therefore, we have developed a multimode coupled *k*_ic_ formalism including the Duschinsky rotation effect (DRE)^7^ (see Part III of ESI†). The Duschinsky rotation matrix (DRM) *M* correlates the normal coordinates of the ground state S_0_ (*Q*_g_) and the excited state S_1_ (*Q*_e_) as *Q*_e_ = *MQ*_g_ + *D*_e_. The DRE occurs most notably for low-frequency modes and becomes more remarkable when more modes with lower frequency are activated, which significantly increases the *k*_ic_ according to our previous investigations.^7^ Hence, the frequency reduction from isotope substitution gives rise to two competitive effects on *k*_ic_: a negative effect through increasing the Huang–Rhys factor and a positive effect *via* strengthening of the DRE to enhance inter-mode mixing (Chart 1). The IE on *k*_ic_ can be defined as,1
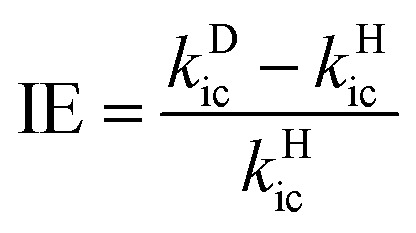
where *k*Dic and *k*Hic are the internal conversion rates for the deuterated and hydrogen system, respectively. As the low-frequency modes are suppressed in the solid/rigid matrix for AIEgens, the DRE becomes minor, and the positive effect diminishes. Then, a more remarkable IE can be anticipated in the solid state compared to in solution. While for the non-AIEgens, since the nonradiative decay is mainly controlled by the vibronic coupling of high-frequency vibration modes with little DRE, the IE is expected to be normal and behaves similarly in the solid state and in solution. In this work, we aim to make such a proposal to use IE for elucidating the microscopic mechanism of the AIE phenomena through theoretical prediction followed by experimental validation.

**Chart 2:**
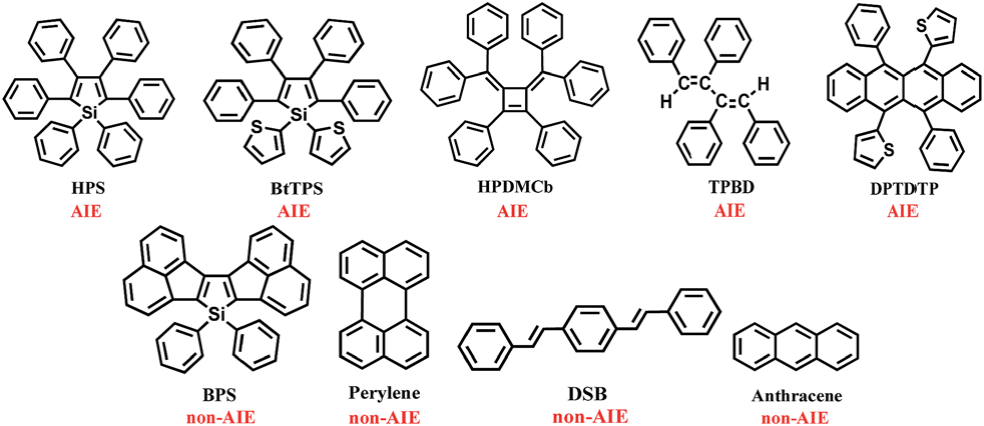
Molecular structures of the AIE and non-AIE compounds.

We investigated the isotopic substitution effects on the luminescent properties in solution and aggregation of the AIE-active 1,1,2,3,4,5-hexaphenylsilole (**HPS**),^8^ 1,1-bis(2′-thienyl)-2,3,4,5-tetraphenylsilole (**BtTPS**),^9^ 1,2-diphenyl-3,4-bis (diphenylmethylene)-1-cyclobutene (**HPDMCb**),^10^*cis*,*cis*-1,2,3,4-tetraphenyl-1,3-butadiene (**TPBD**),^11^ and 2,2′-(6,12-diphenyltetracene-5,11-diyl)dithiophene (**DPTDTP**),^12^ in comparison with AIE-inactive diacenaphtho-[1,2-*b*;1′,2′-*d*]silole (**BPS**),^13^ perylene,^14^ distyrylbenzene (**DSB**),^15^ and anthracene^14^ (see Chart 2).

## Results and discussion

**Fig. 1:**
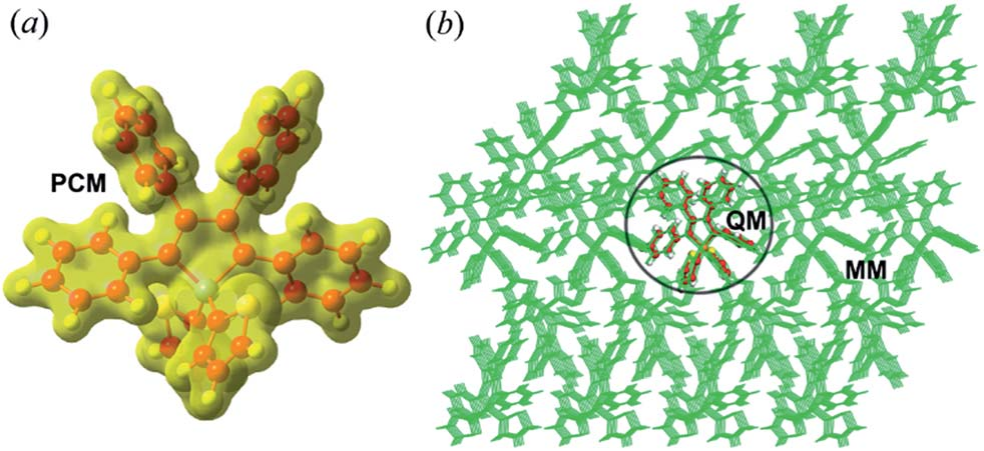
Setup of PCM (a) and QM/MM (b) models (taking **BtTPS** as an example).

The equilibrium geometries and frequencies at the S_0_(S_1_) state of the compounds were calculated at the (TD)-DFT/PBE0/6-31G(d) level, which has been proven to be an effective method to study the electronic structures of the organic molecules.^16^ The environmental effects are taken into account by employing a polarizable continuum model (PCM)^17^ for the solution phase and hybrid quantum mechanics/molecular mechanics (QM/MM)^18^ approach for the aggregates (computational models are given in Fig. 1 and Part I of ESI†). To better match the experimental absorption and emission maxima, we adopted the long-range separated CAM-B3LYP functional to recalculate the excitation energy of the optimized structure. The different PCM models, such as the linear-response (LR)19*a* and state-specific (SS)19*b* solvation methods were tested (see Part II and Tables S1–S9 of ESI†). As expected, the SS-PCM model performs better in describing the excitation energy, probably owing to the self-consistency between the solvent reaction field and the solute electrostatic potential, and has been widely used to investigate the optical properties of solution-phase organic molecules.^20^,^21^ The resultant vertical excitation energies and the comparisons between experimental and theoretical values are given in Table 1, and all deviations are within the acceptable range of 0.01–0.43 eV.^22^

**Table 1:** Calculated optical spectral properties (eV) for the AIE-active and AIE-inactive compounds in both solution and solid phases, as well as the experimental values in parenthesis for comparison

	**HPS**		**BtTPS**		**HPDMCb**	
	Solution	Solid	Solution	Solid	Solution	Solid
Abs.	3.44 (3.39)^*a*^	3.39 (3.36)^*b*^	3.43 (3.33)^*d*^	3.44 (3.36)^*d*^	3.66 (—)	3.61 (3.50)^*e*^
Emi.	2.38 (2.48)^*a*^	2.51 (2.68)^*c*^	2.33 (—)	2.48 (2.59)^*d*^	2.38 (—)	2.49 (2.62)^*e*^

	**TPBD**		**DPTDTP**		**BPS**	
	Solution	Solid	Solution	Solid	Solution	Solid
Abs.	3.64 (3.71)^*f*^	3.83 (3.60)^*f*^	2.35 (2.34)^*g*^	2.37 (2.30)^*g*^	2.12 (2.14)^*h*^	2.07 (—)
Emi.	2.61 (3.04)^*f*^	3.09 (3.18)^*f*^	1.79 (—)	2.04 (2.14)^*g*^	1.54 (—)	1.51 (—)

	**Perylene**		**DSB**		**Anthracene**	
	Solution	Solid	Solution	Solid	Solution	Solid
Abs.	2.99 (2.88)^*i*^	2.99 (2.66)^*j*^	3.32 (3.32)^*k*^	3.32 (—)	3.37 (3.32)^*m*^	3.37 (3.18)^*n*^
Emi.	2.65 (2.82)^*i*^	2.66 (2.61)^*j*^	2.85 (3.18)^*k*^	2.91 (2.72)^*l*^	2.92 (3.30)^*m*^	2.93 (2.93)^*n*^

^*a*^In acetone.^8^

^*b*^In thin film.23*a*

^*c*^In crystal.23*b*

^*d*^In acetone and crystal.^9^

^*e*^In crystal.^10^

^*f*^In acetone and aggregate.^11^

^*g*^In THF and aggregate.^12^

^*h*^In CH_2_Cl_2_.^13^

^*i*^In cyclohexane.23*c*

^*j*^In crystal.^14^

^*k*^In CH_2_Cl_2_.^15^

^*l*^In crystal.^15^

^*m*^In cyclohexane.23*d*

^*n*^In crystal.^14^

We assume the deuterium substitutions hardly change the crystal structure and neglect the induced effects caused by environmental (MM part) deuteration. Normal mode analyses before and after deuteration were performed with the help of the DUSHIN^24^ program. Finally, we evaluated *k*_ic_ using the multimode coupled thermal vibration correlation function formalism realized in the home-built MOMAP program,^25^ which has successfully described the optical properties of many polyatomic molecules, including AIEgens and non-AIEgens.^26^ Both the distortion and DRE of the potential energy surfaces are taken into account in the *k*_ic_ formula (see Part III of ESI†).

**Fig. 2:**
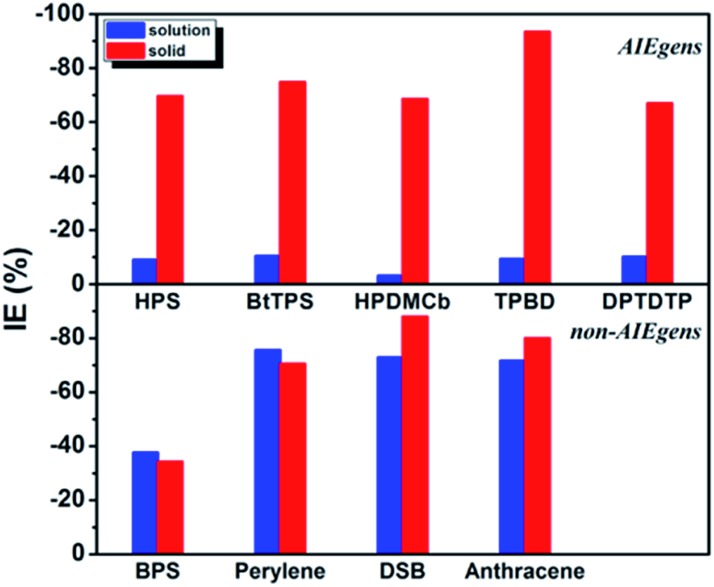
IE results for AIEgens and non-AIEgens.

The calculated results for *k*_ic_ as well as the IE results are presented in Table 2. It is seen that the *k*_ic_ decreases sharply by several orders of magnitude from the solution to solid phases for AIEgens but undergoes a slight change for non-AIEgens. We then plotted the IE results as shown in Fig. 2. It is clear that the IE is always negative. For the AIEgens, the IE is minor (less than *ca.* –10%) in solution but becomes remarkable in a solid phase (*ca.* –65% to –95%). Interestingly, the IE is strikingly different for the AIEgens compared to the non-AIEgens. For non-AIEgens, the IE results both in solution and solid phase are close to each other and fall within the range of *ca.* –40% to –90%.

**Table 2:** Calculated room-temperature *k*_ic_ (s^–1^) for non-deuterated (H-all) and fully-deuterated (D-all) isotopomers of the AIEgens and non-AIEgens in both solution and solid phases

	**HPS**		**BtTPS**		**HPDMCb**	
	Solution	Solid	Solution	Solid	Solution	Solid
H-all	2.44 × 10^11^	8.60 × 10^6^	2.20 × 10^11^	2.73 × 10^7^	1.31 × 10^11^	2.26 × 10^7^
D-all	2.22 × 10^11^	2.61 × 10^6^	1.97 × 10^11^	6.89 × 10^6^	1.27 × 10^11^	7.11 × 10^6^
IE	–9.0%	–69.6%	–10.5%	–74.8%	–3.1%	–68.5%

	**TPBD**		**DPTDTP**		**BPS**	
	Solution	Solid	Solution	Solid	Solution	Solid
H-all	2.16 × 10^10^	4.21 × 10^6^	1.79 × 10^9^	6.76 × 10^5^	1.13 × 10^10^	2.19 × 10^9^
D-all	1.96 × 10^10^	2.77 × 10^5^	1.61 × 10^9^	2.23 × 10^5^	7.05 × 10^9^	1.44 × 10^9^
IE	–9.3%	–93.4%	–10.1%	–67.0%	–37.6%	–34.2%

	**Perylene**		**DSB**		**Anthracene**	
	Solution	Solid	Solution	Solid	Solution	Solid
H-all	1.19 × 10^3^	0.61 × 10^3^	3.84 × 10^3^	5.51 × 10^3^	0.81 × 10^3^	5.25 × 10^3^
D-all	0.29 × 10^3^	0.18 × 10^3^	1.04 × 10^3^	0.66 × 10^3^	0.23 × 10^3^	1.05 × 10^3^
IE	–75.6%	–70.5%	–72.9%	–88.0%	–71.6%	–80.0%

These results can be well justified under the RIM mechanism. Since the internal conversion rate *k*_ic_ depends exponentially on –*S*_*j*_, lowering *ω*_*j*_ implies an increased vibronic coupling strength since the total reorganization energy is unchanged upon deuteration. Thus, the negative IE on *k*_ic_ is generally considered as normal behavior. But for AIEgens in solution, the DRE caused by the low-frequency mode mixing plays a very important role in increasing the internal conversion rate.^7^ Lessening the frequency by isotope substitutions leads to a more pronounced DRE, which tends to sharply increase *k*_ic_ in solution. This counteracts the general IE-induced decrease in *k*_ic_ to a large extent. Thus, full deuteration leads to a much lesser IE for AIEgens in solution, distinct from AIEgens in solid phase and non-AIEgens in solution or solid phases.

**Fig. 3:**
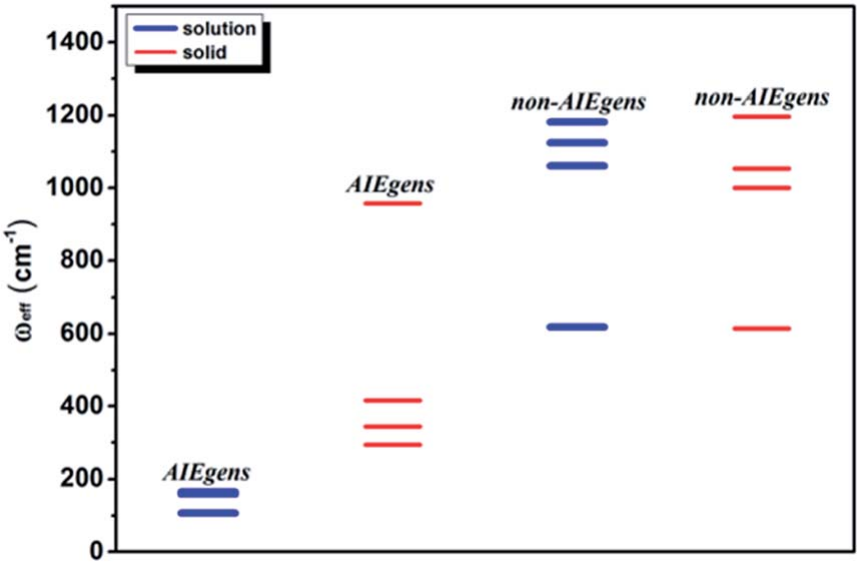
*ω*_eff_ for AIEgens and non-AIEgens in both solution and solid phases.

We further introduce an effective frequency (*ω*_eff_) for a more quantitative interpretation,^27^2
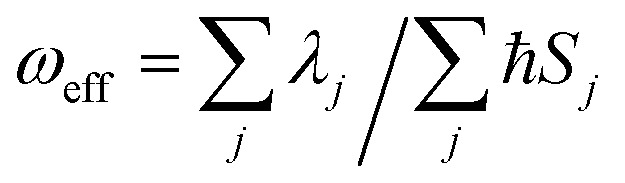
which represents an effective mode contributing to the excited-state relaxation process. The *ω*_eff_ results for the pristine systems are shown in Fig. 3. It is noted that the *ω*_eff_ for AIEgens falls into the range of 100–160 cm^–1^ in solution since the low-frequency modes (<100 cm^–1^) contribute largely to the relaxation energy. But upon aggregation, the *ω*_eff_ rises to 300–1000 cm^–1^ owing to the dominating high frequency vibrations. The *ω*_eff_ (600–1200 cm^–1^) is close for all the non-AIEgens in solution and solid states. In addition, the deuteration effect for *ω*_eff_ is given in Table S10† and the *ω*_eff_ always decreases as expected. Detailed analyses of vibrational and structural relaxation modification induced by aggregation are presented in Part IV, Fig. S1–S7 and Tables S11–S18 of the ESI.†

**Fig. 4:**
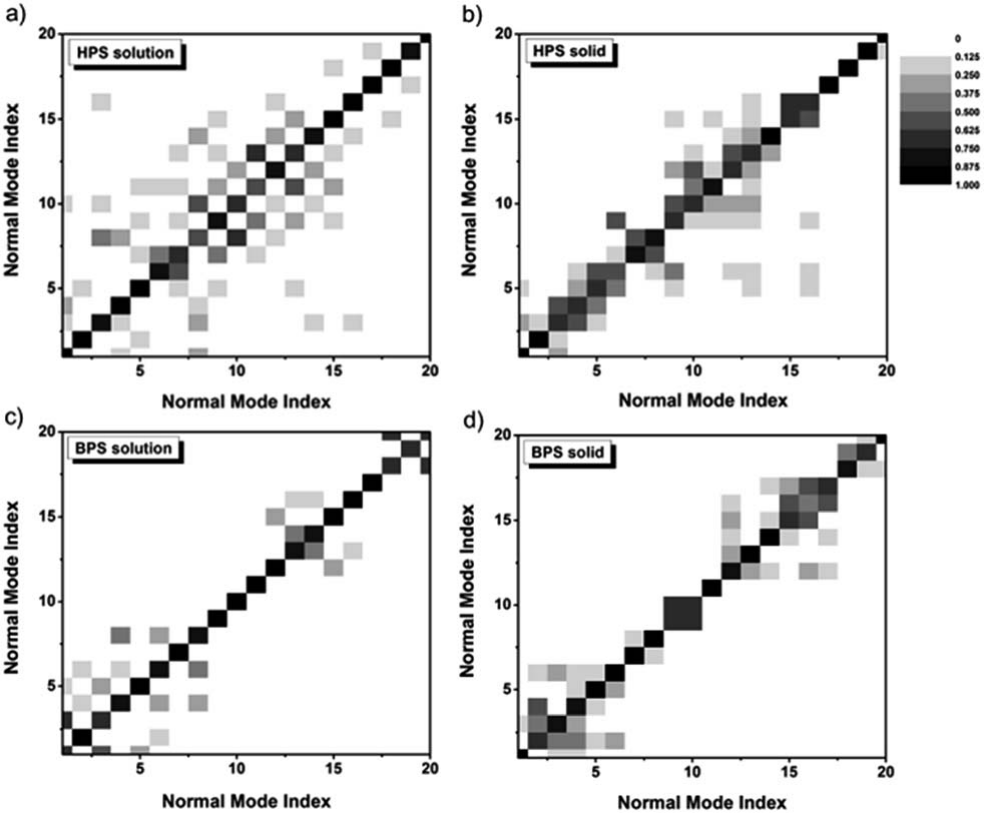
Contour maps of the DRM for the lowest 20 modes in **HPS** solution (a), **HPS** solid phase (b), **BPS** solution (c), and **BPS** solid phase (d). The excited-state normal mode index is rearranged to make the largest element the diagonal.

The difference of the IE for solution-phase AIEgens compared to other cases mainly stems from the outstanding contributions of low-frequency normal modes during the excited-state decay process. The low-frequency modes arise from the flat potential energy surfaces, and the involved quantum number of these modes increases sharply with temperature. At room temperature, there are so many vibrational states which are close in energy. They tend to strongly mix among the different modes, namely, the DRE becomes very pronounced, leading to the increase of *k*_ic_. The DRE can be directly characterized from the calculated DRM. The more the diagonal elements of the DRM deviate from 1.0, the more off-diagonal elements are non-zero, and the more significant the DRE is. The contour maps of the DRM for 20 normal modes with the lowest frequencies are presented in Fig. 4. For both solution-phase and solid-phase non-AIEgens, *e.g.***BPS**, most diagonal elements are close to 1.0 and the DRE is tiny. A similar situation can be found for the AIEgens in the solid-phase *e.g.* for **HPS**, most of the lowest 20 modes are in the range of 100–200 cm^–1^ and the DRM elements gather in the vicinity of the diagonal line, indicating a very weak DRE. However, for AIEgens in the solution phase, the off-diagonal elements of the DRM become more outstanding corresponding to more considerable mixing between modes. When the frequencies of the modes decrease upon deuteration, the effect would become much more severe, this could sharply increase the *k*_ic_ and compensate for the normal negative IE on *k*_ic_. To validate this assumption, we also calculate *k*_ic_ without the DRE for a typical AIEgen, **HPS**, and non-AIEgen, **BPS**, in both solution and solid phases (see Table S19†). IEs without the DRE are –88.4% (–71.4%) for **HPS** in solution (solid phase) and –47.8% (–48.0%) for **BPS** in solution (solid phase), respectively. When the DRE is considered, it is only –9.0% for solution-phase **HPS**. Therefore, isotopic substitution could be utilized as an effective tool to explore the aggregation effect on the nonradiative process. The DRE suppressed by molecular aggregation in AIEgens could result in a sudden “jump” or “drop” of the IE.

To experimentally verify the above calculated isotopic characteristic of AIEgens, we synthesized 2,3,4,5-ring deuterated (D-) **HPS** (see Part V of the ESI†) and measured the radiative and nonradiative decay rates for a comparison with the pristine **HPS** as presented in Table 3. The experimental values are in good agreement with the calculated ones (computational details presented in Part VI and Table S20 of the ESI†). In solution, both the H- **HPS** and D- **HPS** are non-emissive as determined from both the calculated and experimental results because the nonradiative decay rates are far larger than the radiative ones, while they emit strong light in the solid state. Strikingly, the experimental IEs well reproduce the calculated results, in that they are very little in solution but remarkable in solid phase. In particular, the abnormal positive effect of the IE is observed in the experiment, which fully confirms the essential role of the DRE in the nonradiative decay process. In solid phase, a large normal negative IE is observed because the nonradiative decay rate is mainly determined by the vibronic coupling.

**Table 3:** The calculated and the measured excited-state decay rates of the non-deuterated (H-) and deuterated (D-)2,3,4,5-ring **HPS** in solution and solid phases at room temperature

**HPS**	Solution			Solid		
	*k*_r_	*k*_ic_	IE	*k*_r_	*k*_ic_	IE
H-	6.54 × 10^7^ ^*a*^	2.44 × 10^11^ ^*a*^		0.83 × 10^7^ ^*c*^	0.86 × 10^7^ ^*c*^	
	(1.27 × 10^7^)^*b*^	(1.05 × 10^9^)^*b*^		(1.52 × 10^7^)^*d*^	(1.29 × 10^7^)^*d*^	
D-	6.54 × 10^7^ ^*a*^	2.30 × 10^11^ ^*a*^	–5.7%	0.83 × 10^7^ ^*c*^	0.29 × 10^7^ ^*c*^	–66.3%
	(1.64 × 10^7^)^*b*^	(1.07 × 10^9^)^*b*^	(1.9%)^*b*^	(1.37 × 10^7^)^*d*^	(0.47 × 10^7^)^*d*^	(–63.6%)^*d*^

^*a*^Calculated in acetone solution.

^*b*^Measured in THF solution.

^*c*^Calculated in crystal.

^*d*^Measured in microcrystal.

**Fig. 5:**
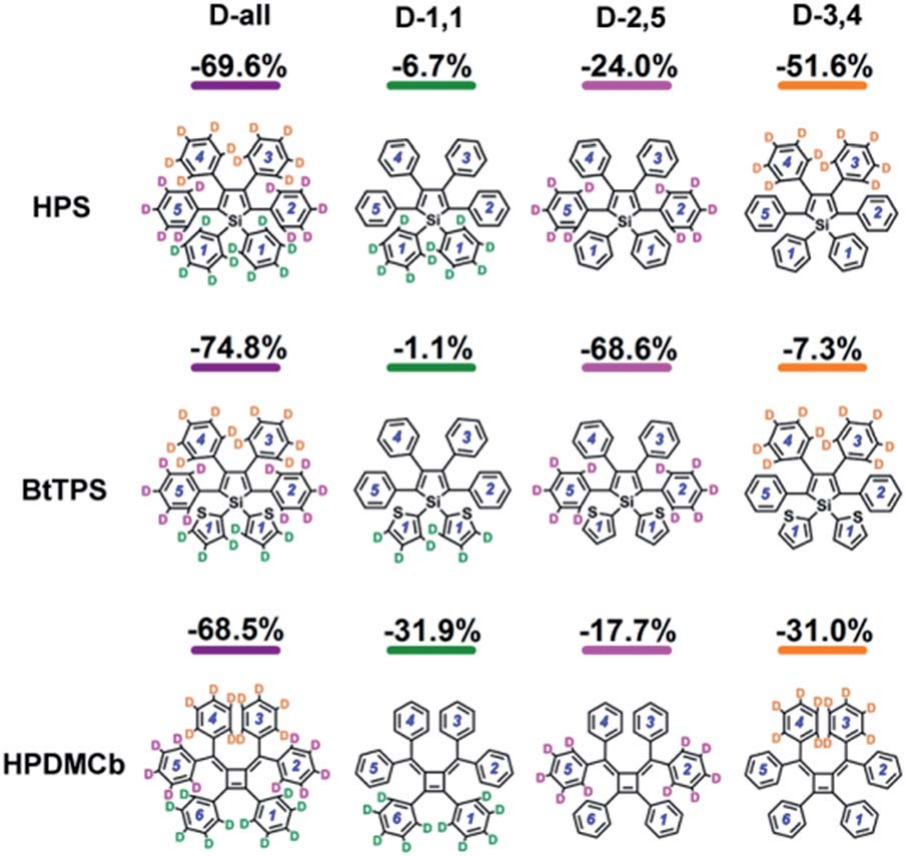
IEs for all deuterated isotopomers of **HPS**, **BtTPS** and **HPDMCb** in solid phase.

We further systematically investigated the isotope substituted position dependence as to provide more experimentally verifiable quantitative predictions, taking the 6-ring analogues **HPS**, **BtTPS**, and **HPDMCb** as examples. Partially-deuterated schemes are considered in both solution and solid phases, namely, 1,1-rings or 1,6-rings only deuterated (D-1,1), 2,5-rings only deuterated (D-2,5), and 3,4-rings only deuterated (D-3,4). Detailed data are presented in Part VII and Table S21 of the ESI.† It is obvious that the IEs in solid states are more remarkable than those in solution. We herein analyse the cases in solid phase as illustrated in Fig. 5. For **HPS**, the IE follows the order of D-3,4 > D-2,5 > D-1,1; for **BtTPS**, D-2,5 induces a much larger IE than D-3,4 and D-1,1; **HPDMCb** also shows position-dependent deuteration effects, with a major IE induced by D-1,1 and D-3,4 but a minor IE induced by D-2,5. Interestingly, the IE decreases in the order of the reduction of *ω*_eff_ (see Table S22†) for the partially deuterated isotopomers, which further confirms the *ω*_eff_ to be a good parameter to judge the IE. *E.g.* for **BtTPS**, D-2,5 undergoes a remarkable frequency decrease (–3.5%), while D-3,4 experiences a slight frequency change (–0.6%), and D-1,1 exhibits an almost unaffected frequency (–0.3%).

**Fig. 6:**
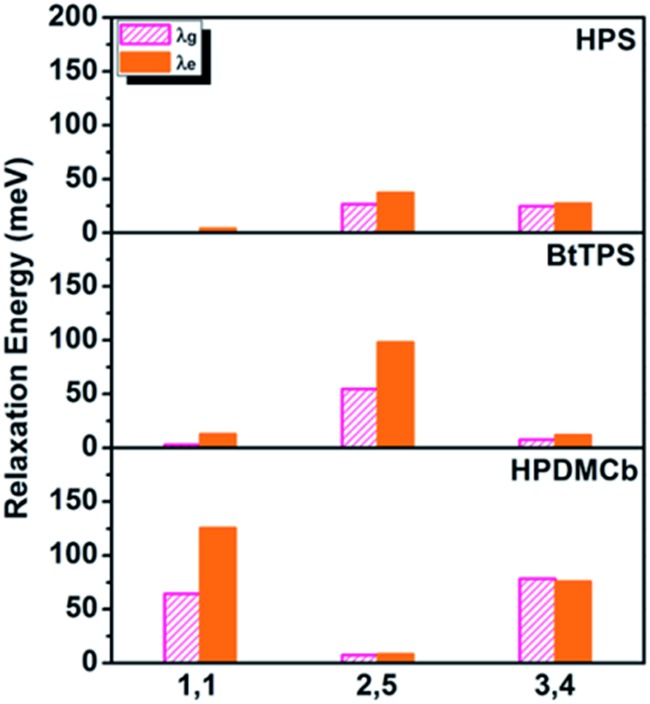
Position dependent relaxation energy for solid-phase **HPS**, **BtTPS** and **HPDMCb**.

Deuteration influences mostly the C–H bond stretching and C–H in-plane or out-of-plane bending vibrations. For complex polyatomic molecules, these vibrations are naturally mixed with other vibrations such as CCC in-plane bending, CC stretching or ring out-of-plane deformation or twisting vibration (see Tables S13 and S17†). The deuteration effect becomes non-trivial only when the corresponding relaxation energy is important. The vibration types of significant modes with major relaxation energies (see Fig. S2 and S8†) are detailed in Fig. S4, S9 and S10 and Tables S13 and S23.† *E.g.* for **BtTPS**, these vibrations are mainly from 2,5-rings, with minor contributions from 1,1,3,4-rings. To elaborate this more clearly, we project the total relaxation energy onto the geometry relaxation in internal coordinates. The contributions from the internal coordinates of the rings at the 1,1-positions or 1,6-positions (1,1), 2,5-positions (2,5) and 3,4-positions (3,4) to the total relaxation energy are depicted in Fig. 6 and listed in Tables S24–26.† It is clearly seen that the substitution positions contributing largely to the relaxation energy could induce remarkable deuteration effects, *i.e.*, for **HPS**, D-3,4 and D-2,5 have obvious effects; for **BtTPS**, D-2,5 stands out; for **HPDMCb**, D-1,1 and D-3,4 have remarkable effects.

## Conclusions

In summary, we investigated the isotope effect on the excited-state nonradiative decay rate for a series of AIEgens in both solution and solid phases in comparison with non-AIEgens through first-principles calculations. We found that deuteration always leads to a negative IE on the nonradiative decay rate in both solution and solid phases as expected. Interestingly, the IE is inconspicuous (*ca.* –10%) for AIEgens in solution in sharp contrast to that of the solid phase of *ca.* –65% to –95%. The deuteration effect has been observed in the experiments of a typical AIEgen, **HPS**, in solution and solid states. For non-AIEgens, the IE is comparable (*ca.* –40% to –90%) in solution and in solid states. The minor IE for AIEgens in solution stems from the competitive results of the negative effect induced by an increased vibronic coupling strength characterized by the Huang–Rhys factor and a positive effect caused by an enhanced DRE. The DRE mainly occurs for low-frequency rotating or twisting vibrations and it becomes weak for AIEgens in solid state compared to in solution. Therefore the IEs of AIEgens in solid phases are similar to those of non-AIEgens in solution and solid states because the nonradiative decay rates in these cases are mainly controlled by the vibronic coupling. Thus, isotopic substitution is shown to be a valuable tool to elucidate the nonradiative decay mechanism induced by aggregation. Partial deuteration effects are estimated for the 6-ring AIE analogues to evaluate the positional dependence. The positions involved in the vibrations with large relaxation energy are found to be responsible for the dependence. Agreement between the theoretical calculations and experimental measurements validates the RIM mechanism for AIE phenomena.

Finally, we should note that computational studies of excited-state dynamics are full of challenges.^28^ The present methodology focuses only on the intramolecular process by assuming a simple displaced and distorted harmonic oscillator model. As has been shown previously,26*a* the vibrational quanta decrease steadily with the molecular size. At the same time, the electronic adiabatic transition energy also decreases with size, implying a reduced anharmonic effect for a large system. For further developments by considering the anharmonicity, and excitonic effect as well as charge transfer delocalization, non-perturbation, *etc.* on the spectroscopy and nonradiative decay rates of multichromophoric aggregates, there is still a long way to go toward the quantitative prediction of light-emitting properties based on first-principles.

## Experimental

### *Ab initio* calculations

The solution-phase geometry optimizations and frequency calculations were performed through the equilibrium solvation method. Vertical transition energies at the optimized geometries were obtained *via* the non-equilibrium solvation method. Solid-phase geometry optimizations were performed on the QM region while the MM region was kept frozen. The centroid molecule of the cluster built from the X-ray crystal structure was chosen as the QM region, whereas the remaining molecules were treated as the MM region. The excitonic effect as well as the intermolecular charge transfer was ignored, since AIEgens in general present twisted structures with large intermolecular distances (more than 5 Å) without π–π interactions.16c,26c,26d For non-AIEgens, flat molecular planes tend to favor π–π stacking interactions, where the radiative process could be strongly affected by forming H- or J-aggregates or excimers. Moreover, the Coulomb coupling components of the excitonic couplings for the representative **HPS** and **BPS** were calculated (see Part VIII of the ESI†). The intermolecular excitonic couplings for **HPS** and **BPS** are at least one order of magnitude smaller than the intramolecular relaxation energy (Fig. S11 and S12 and Tables S27 and S28†), implying that the photophysical process is dominated by intramolecular motions. No symmetry constraint was imposed during the optimizations in both solution and solid phases. The absence of imaginary frequencies was carefully checked.

### Materials and instruments

The absolute fluorescence quantum yields were determined by a calibrated integrating sphere of a Hamamatsu absolute PL quantum yield spectrometer C11347 Quantaurus-QY, excitation wavelength: 365 nm. Spectrophotometric-grade solvents were used in the measurements without further purification.

## Supplementary Material

Supplementary information
