# Supplementary material for: Using the isotope effect to probe an aggregation induced emission mechanism: theoretical prediction and experimental validation
Source: Chem Sci. 2016 May 11;7(8):5573–80. doi: 10.1039/c6sc00839a (PMC6022082; doi:10.1039/c6sc00839a)
Supplement: Supplementary file 1 [file SC-007-C6SC00839A-s001.pdf]

Electronic Supplementary Material (ESI) for Chemical Science.
This journal is © The Royal Society of Chemistry 2016

Electronic Supplementary Information for

Using the isotope effect to probe an aggregation induced emission mechanism: theoretical prediction and experimental validation

Tian Zhang,[a] Qian Peng,[*b] Changyun Quan,[c] Han Nie,[c] Yingli Niu,[d] Yujun Xie,[e] Zujin Zhao,[c] Ben Zhong Tang[c] and Zhigang Shuai[*a,f]

[a] Key Laboratory of Organic OptoElectronics and Molecular Engineering, Department of Chemistry, Tsinghua University, Beijing, 100084, China. E-mail: zgshuai@tsinghua.edu.cn

[b] Key Laboratory of Organic Solids, Beijing National Laboratory for Molecular Science (BNLMS), Institute of Chemistry, Chinese Academy of Sciences, Beijing, 100190, China. E-mail: qpeng@iccas.ac.cn

[c] State Key Laboratory of Luminescent Materials and Devices, South China University of Technology, Guangzhou, 510640, China

[d] National Center for Nanoscience and Technology, Chinese Academy of Sciences, Beijing, 100190, China

[e] Department of Chemistry, Wuhan University, Wuhan, 430072, China

[f] Collaborative Innovation Center of Chemistry for Energy Materials, Xiamen University, Xiamen, 351005, China

Contents

I. Computational Details of PCM and QM/MM

II. Benchmark on the Vertical Excitation Energies

III. Theoretical Formalism for k_{ic}

IV. Normal Mode Analysis and Geometric/Electronic Structure for **HPS** and **BPS**

V. Synthesis of H- and D- **HPS**

VI. Computation for k_r and k_{ic} of H- and D- **HPS**

VII. Partial Deuteration Effects for the 6-ring AIE analogues

VIII. Excitonic Couplings for **HPS** and **BPS**

I. Computational Details of PCM and QM/MM

PCM is implemented in the D.01 version of Gaussian 09[S1] package. **HPS**, **BtTPS** and **TPBD** were modeled in acetone solution, **HPDMCb** was modeled in acetonitrile solution, **DPTDTP** was modeled in tetrahydrofuran (THF) solution, **BPS** and **DSB** were modeled in dichloromethane (CH_2Cl_2) solution, **Perylene** and **Anthracene** were modeled in cyclohexane solution. We also note the recent development of the explicit solvation model, *i.e.*, real-time approaches[S2] and excited-state *ab initio* molecular dynamics[S3], taking the fluctuations of the bulk solvent into account. The QM/MM energy and gradient calculations were interfaced with the ChemShell 3.5[S4] program. The QM/MM electrostatic interaction is evaluated with Turbomole 6.5[S5], incorporating the MM partial charges into the effective Hamiltonian of the QM part.[S6] The QM/MM van der Waals (vdW) interaction is managed by DL_POLY[S7] via Lenard-Jones formalism. The general Amber force field (GAFF)[S8] was adopted for the force-field expressions. The vibrational frequencies were obtained with the numerical two-point displacement approach. Translational and rotational spaces were projected out for all Hessian calculations through Gram-Schmidt orthogonalization.[S9] The QM/MM models for **HPS**, **BtTPS**, **HPDMCb**, **TPBD**, **DPTDTP**, **DCPP**, **BPS**, **Perylene**, **DSB**, **Anthracene** were built in the following:

(1) for **HPS**, a cluster of 75 molecules consisting of 71 QM atoms and 5254 MM atoms is built from the X-ray crystal structure[8]

(2) for **BtTPS** , a cluster of 105 molecules consisting of 65 QM atoms and 6760 MM atoms is built from X-ray crystal structure[9]

(3) for **HPDMCb,** a cluster of 65 molecules consisting of 72 QM atoms and 4608 MM atoms is built from X-ray crystal structure[10]

(4) for **TPBD**, a cluster of 65 molecules consisting of 50 QM atoms and 3200 MM atoms is built

from X-ray crystal structure[S10]

(5) for **DPTDTP**, a cluster of 51 molecules consisting of 64 QM atoms and 3200 MM atoms is built from X-ray crystal[12]

(6) for **BPS** a cluster of 72 molecules consisting of 59 QM atoms and 4189 MM atoms is built from X-ray crystal[13]

(7) for **Perylene**, a cluster of 36 molecules consisting of 32 QM atoms and 1120 MM atoms is built from X-ray crystal[S11]

(8) for **DSB**, a cluster of 48 molecules consisting of 40 QM atoms and 1880 MM atoms built from X-ray crystal[15]

(9) for **Anthracene** a cluster of 57 molecules consisting of 24 QM atoms and 1344 MM atoms is built from X-ray crystal[S12]

II. Benchmark on the Vertical Excitation Energies

The excited state energy for the 9 compounds are calculated and benchmarked with the experimental values (Tables S1-S9). Eventually, CAM-B3LYP/SS-PCM//PBE0/LR-PCM and CAM-B3LYP/QM-MM//PBE0/QM-MM schemes were adopted to describe the transition properties for **HPS**, **BtTPS** and **HPDMCb** in solution and solid, respectively. PBE0/SS-PCM//PBE0/LR-PCM and PBE0/QM-MM schemes were chosen to treat **TPBD**, **DPTDTP**, **BPS**, **Perylene**, **DSB**, **Anthracene** since CAM-B3LYP substantially overestimates the vertical excitation energies. The lowest excited singlet states of all systems investigated in this work mainly stem from the single excitation from HOMO to LUMO in both solution and solid phases, with the component more than 95% , indicating the suitability to employ TD-DFT method.[S13]

Table S1 The vertical transition energies for solution-phase **HPS** calculated by using methods of PBE0/LR-PCM, PBE0/SS-PCM, CAM-B3LYP/LR-PCM and CAM-B3LYP/SS-PCM based on the PBE0/LR-PCM optimized $\mathbf{S_0}$ and $\mathbf{S_1}$ structures. The vertical transition energies for solid-phase **HPS**

calculated by using methods of PBE0/QMMM, CAM-B3LYP/QMMM based on the PBE0/QMMM optimized $\mathbf{S_0}$ and $\mathbf{S_1}$ structures. The experimental values (expt.) are also given.

	absorption			emission		
	PBE0	CAM-B3LYP	expt.	PBE0	CAM-B3LYP	expt.
LR-PCM	3.12 eV	3.39 eV		2.08 eV	2.18 eV	
	(398 nm)	(366 nm)	3.39 eV	(596 nm)	(568 nm)	2.48 eV
SS-PCM	3.16 eV	3.44 eV	(366 nm)	2.23 eV	2.38 eV	(499 nm)
	(392 nm)	(361 nm)		(555 nm)	(522 nm)	
QM/MM	3.14 eV	3.39 eV	3.36 eV	2.37 eV	2.51 eV	2.68 eV
	(395 nm)	(366 nm)	(369 nm)	(522 nm)	(494 nm)	(462 nm)

Table S2 The vertical transition energies for solution-phase **BtTPS** calculated by using methods of PBE0/LR-PCM, PBE0/SS-PCM, CAM-B3LYP/LR-PCM and CAM-B3LYP/SS-PCM based on the PBE0/LR-PCM optimized $\mathbf{S_0}$ and $\mathbf{S_1}$ structures. The vertical transition energies for solid-phase **BtTPS** calculated by using methods of PBE0/QMMM, CAM-B3LYP/QMMM based on the PBE0/QMMM optimized $\mathbf{S_0}$ and $\mathbf{S_1}$ structures. The experimental values (expt.) are also given.

	absorption			emission		
	PBE0	CAM-B3LYP	expt.	PBE0	CAM-B3LYP	expt.
LR-PCM	3.10 eV	3.39 eV		2.04 eV	2.29 eV	
	(400 nm)	(366 nm)	3.33 eV	(609 nm)	(541 nm)	N. A.
SS-PCM	3.14 eV	3.43 eV	(372 nm)	2.17 eV	2.33 eV	
	(395 nm)	(361 nm)		(571 nm)	(532 nm)	
QM/MM	3.13 eV	3.44 eV	3.36 eV	2.29 eV	2.48 eV	2.59 eV
	(396 nm)	(360 nm)	(369 nm)	(541 nm)	(500 nm)	(478 nm)

Table S3 The vertical transition energies for solution-phase **HPDMCb** calculated by using methods of PBE0/LR-PCM, PBE0/SS-PCM, CAM-B3LYP/LR-PCM and CAM-B3LYP/SS-PCM based on the PBE0/LR-PCM optimized $\mathbf{S_0}$ and $\mathbf{S_1}$ structures. The vertical transition energies for solid-phase **HPDMCb** calculated by using methods of PBE0/QMMM, CAM-B3LYP/QMMM based on the PBE0/QMMM optimized $\mathbf{S_0}$ and $\mathbf{S_1}$ structures. The experimental values (expt.) are also given.

	absorption			emission		
	PBE0	CAM-B3LYP	expt.	PBE0	CAM-B3LYP	expt.
LR-PCM	3.26 eV	3.62 eV	N. A.	2.09 eV	2.37 eV	N. A.
	(381 nm)	(342 nm)		(592 nm)	(523 nm)	
SS-PCM	3.15 eV	3.66 eV		2.09 eV	2.38 eV	
	(393 nm)	(339 nm)		(594 nm)	(521 nm)	
QM/MM	3.24 eV	3.61 eV	3.50 eV	2.27 eV	2.49 eV	2.62 eV
	(383 nm)	(343 nm)	(354 nm)	(547 nm)	(497 nm)	(474 nm)

Table S4 The vertical transition energies for solution-phase **TPBD** calculated by using methods of PBE0/LR-PCM, PBE0/SS-PCM based on the PBE0/LR-PCM optimized $\mathbf{S_0}$ and $\mathbf{S_1}$ structures and for solid-phase BPS at the PBE0/QMMM level. The experimental values (expt.) are also given.

	absorption		emission	
	PBE0	expt.	PBE0	expt.
LR-PCM	3.53 eV		2.29 eV	
	(351 nm)	3.71 eV	(542 nm)	3.04 eV
SS-PCM	3.64 eV	(334 nm)	2.61 eV	(408 nm)
	(341nm)		(475 nm)	
QM/MM	3.83 eV	3.60 eV	3.09 eV	3.18 eV
	(323 nm)	(344 nm)	(401 nm)	(390 nm)

Table S5 The vertical transition energies for solution-phase **DPTDTP** calculated by using methods of PBE0/LR-PCM, PBE0/SS-PCM based on the PBE0/LR-PCM optimized $\mathbf{S_0}$ and $\mathbf{S_1}$ structures and for solid-phase BPS at the PBE0/QMMM level. The experimental values (expt.) are also given.

	absorption		emission	
	PBE0	expt.	PBE0	expt.
LR-PCM	2.32 eV		1.70 eV	
	(534 nm)	2.34 eV	(729 nm)	N. A.
SS-PCM	2.35 eV	(530 nm)	1.79 eV	
	(527 nm)		(692 nm)	
QM/MM	2.37 eV	2.30 eV	2.04 eV	2.14 eV
	(523 nm)	(540 nm)	(607 nm)	(580 nm)

Table S6 The vertical transition energies for solution-phase **BPS** calculated by using methods of PBE0/LR-PCM, PBE0/SS-PCM based on the PBE0/LR-PCM optimized $\mathbf{S_0}$ and $\mathbf{S_1}$ structures and for solid-phase BPS at the PBE0/QMMM level. The experimental values (expt.) are also given.

	absorption		emission	
	PBE0	expt.	PBE0	expt.
LR-PCM	2.07 eV		1.44 eV	
	(600 nm)	2.14 eV	(859 nm)	N. A.
SS-PCM	2.12 eV	(580 nm)	1.54 eV	
	(586 nm)		(803 nm)	
QM/MM	2.07 eV	N. A	1.51 eV	N. A.
	(600 nm)		(823 nm)	

Table S7 The vertical transition energies for solution-phase **Perylene** calculated by using methods of PBE0/LR-PCM, PBE0/SS-PCM based on the PBE0/LR-PCM optimized $\mathbf{S_0}$ and $\mathbf{S_1}$ structures and for solid-phase BPS at the PBE0/QMMM level. The experimental values (expt.) are also given.

	absorption		emission	
	PBE0	expt.	PBE0	expt.
LR-PCM	2.90 eV		2.65 eV	
	(428 nm)	2.88 eV	(469 nm)	2.82 eV
SS-PCM	2.99 eV	(430 nm)	2.65 eV	(440 nm)
	(415 nm)		(469 nm)	

QM/MM	2.99 eV (414 nm)	2.66 eV (466 nm)	2.66 eV (466 nm)	2.61 eV (475 nm)

Table S8 The vertical transition energies for solution-phase **DSB** calculated by using methods of PBE0/LR-PCM, PBE0/SS-PCM based on the PBE0/LR-PCM optimized $\mathbf{S_0}$ and $\mathbf{S_1}$ structures and for solid-phase BPS at the PBE0/QMMM level. The experimental values (expt.) are also given.

	absorption		emission	
	PBE0	expt.	PBE0	expt.
LR-PCM	3.18 eV		2.51 eV	
	(390 nm)	3.32 eV	(493 nm)	3.18 eV
SS-PCM	3.32 eV	(373 nm)	2.85 eV	(390 nm)
	(373 nm)		(435 nm)	
QM/MM	3.32 eV	N. A.	2.91 eV	2.72 eV
	(373 nm)		(426 nm)	(455 nm)

Table S9 The vertical transition energies for solution-phase **Anthracene** calculated by using methods of PBE0/LR-PCM, PBE0/SS-PCM based on the PBE0/LR-PCM optimized $\mathbf{S_0}$ and $\mathbf{S_1}$ structures and for solid-phase BPS at the PBE0/QMMM level. The experimental values (expt.) are also given.

	absorption		emission	
	PBE0	expt.	PBE0	expt.
LR-PCM	3.33 eV		2.88 eV	
	(372 nm)	3.32 eV	(430 nm)	3.30 eV
SS-PCM	3.37 eV	(373 nm)	2.92 eV	(376 nm)
	(368 nm)		(424 nm)	
QM/MM	3.37 eV	3.18 eV	2.93 eV	2.93 eV
	(368 nm)	(390 nm)	(423 nm)	(423 nm)

III. Theoretical Formalism for calculating k_{ic}

Based on Fermi Golden Rule, we apply thermal vibration correlation function formalism to obtain k_{ic}:[7b,25]

$$k_{ic} = \sum_{kl} \frac{1}{\hbar^2} R_{kl} \int_{-\infty}^{\infty} dt \left[e^{i\omega_{if}t} Z_i^{-1} \rho_{ic,kl}(t,T) \right] \tag{S1}$$

More details of the correlation function part $\rho_{ic,kl}(t,T)$ are given in our previous work.[24b] The normal-mode coordinates of g state (Q_g) and e state (Q_e) are correlated through the Duschinsky rotation matrix $\boldsymbol{M}$ with the relationship $Q_e = \boldsymbol{M}Q_g + \boldsymbol{D}_e$, $\boldsymbol{M}$ and $\boldsymbol{D}_e$ can be calculated through

DUSHIN[24] program.

We applied the first-order perturbation theory to compute the electronic coupling $R_{kl} = \langle \Phi_\mathrm{f} | \hat{P}_{\mathrm{f}k} | \Phi_\mathrm{i} \rangle \langle \Phi_\mathrm{i} | \hat{P}_{\mathrm{f}l} | \Phi_\mathrm{f} \rangle$ following Lin.[5a] At the equilibrium position approximately,

$$\langle \Phi_\mathrm{f} | \hat{P}_{\mathrm{f}l} | \Phi_\mathrm{i} \rangle = -i\hbar \langle \Phi_\mathrm{f} | \frac{\partial}{\partial Q_{\mathrm{f}l}} | \Phi_\mathrm{i} \rangle = -i\hbar \frac{\langle \Phi_\mathrm{f}^0 | \partial \hat{U} / \partial Q_{\mathrm{f}l} | \Phi_\mathrm{i}^0 \rangle}{E_\mathrm{i}^0 - E_\mathrm{f}^0} \quad \text{(S2)}$$

where

$$\langle \Phi_\mathrm{f}^0 | \partial \hat{U} / \partial Q_{\mathrm{f}l} | \Phi_\mathrm{i}^0 \rangle = -\sum_\sigma \frac{Z_\sigma \mathrm{e}^2}{\sqrt{M_\sigma}} \sum_{\tau = x,y,z} E_{\mathrm{f}\leftarrow\mathrm{i},\sigma\tau} L_{\sigma\tau,l} \quad \text{(S3)}$$

The transition electric field $E_{\mathrm{f}\leftarrow\mathrm{i},\sigma\tau} = \int \mathrm{d}\boldsymbol{r} \rho_{\mathrm{fi}}^0(\boldsymbol{r}) \frac{\mathrm{e}(r_\tau - R_{\sigma\tau})}{|\boldsymbol{r} - \boldsymbol{R}_\sigma|^3}$ can be computed directly from TD-PBE0 calculation using D.01 version of Gaussian 09[S1] package, and U is the electron-nuclear potential term in the Hamiltonian. Recently Send and Furche also formulated an analytical derivative[S14] in a finite atom-centered basis set, which has been implemented in Turbomole[S5]. We used the former scheme for the computational study.

Table S10 Effective frequencies (ω_{eff}) for non-deuterated (**H-all**) and fully-deuterated (**D-all**) isotopomers of AIE-active **HPS**, **BtTPS**, **HPDMCb**, **TPBD**, **DPTDTP** and AIE-inactive **BPS**, **Perylene**, **DSB**, **Anthracene** in solution and solid phases^a

		ω_{eff}				
		**HPS**	**BtTPS**	**HPDMCb**	**TPBD**	**DPTDTP**
solution	**H-all**	165	163	107	161	159
	**D-all**	155	153	102	147	156
		(–6.1%)	(–6.1%)	(–4.7%)	(–8.7%)	(–1.9%)
solid	**H-all**	416	344	294	416	958
	**D-all**	399	328	281	377	947
		(–4.1%)	(–4.7%)	(–4.4%)	(–9.4%)	(–1.1%)
		**BPS**	**Perylene**	**DSB**	**Anthracene**	
solution	**H-all**	618	1061	1125	1182	
	**D-all**	606	1047	1107	1169	
		(–1.9%)	(–1.3%)	(–1.6%)	(–1.1%)	
solid	**H-all**	614	1054	1000	1197	
	**D-all**	604	1040	967	1185	
		(–1.6%)	(–1.3%)	(–3.3%)	(–1.0%)	

[a]The percentages of frequency reduction in the original frequencies are included in the parentheses.

IV. Normal Mode Analysis and Geometric/Electronic Structure for HPS and BPS

It has been suggested that AIE phenomenon was caused by the restricted intramolecular rotation/vibration through nonradiative relaxation.[7,26] The nonadiabatic electronic coupling, as a pre-factor in the k_{ic} formula (eqn S1), hardly varies when going from solution to solid (Fig. S1). We project the total relaxation energy ($\lambda_{g(e)} = \sum_{j \in g(e)} \lambda_j = \sum_{j \in g(e)} S_j \hbar\omega_j$) to the normal coordinate (Fig. S2). We take AIE-active **HPS** and its non-AIE counterpart **BPS** as an example for normal mode analysis. The contribution of the low-frequency vibrations to the total reorganization energy is largely reduced from 20% (93 meV) in solution to 6% (24 meV) in solid (Tables S11-S12) for **HPS**. The atomic movement patterns of selected 5 modes with significant contributions to the relaxation energy are depicted (Fig. S3-S4 and Table S13). Four orders of magnitude reduction in k_{ic} is found for **HPS** upon aggregation. From the structural relaxation analysis, it is also seen the "active" phenyl rings at the 2,5-positions of **HPS** are largely hindered in solid (Table S14). However, for non-AIEgen **BPS**, contributions from the low-frequency modes (< 100 cm^{-1}) to the total relaxation energy are found to be negligible (Tables S15-S16). The relaxation energy is insensitive to aggregation, which shows none of the nonradiative decay channels are changed. The 5 representative modes for **BPS** are also illustrated (Fig. S5-S6 and Table S17). Correspondingly, all rings in **BPS** are inactive (Table S18).

The electronic density contours of HOMOs and LUMOs, together with HOMO and LUMO energy levels and HOMO-LUMO gaps are also shown in Fig. S7. The more planar and rigid structure of **BPS** enables better electronic communication and results in a quite smaller gap (ca. 2.80 eV) compared to **HPS** (ca. 3.80 eV).

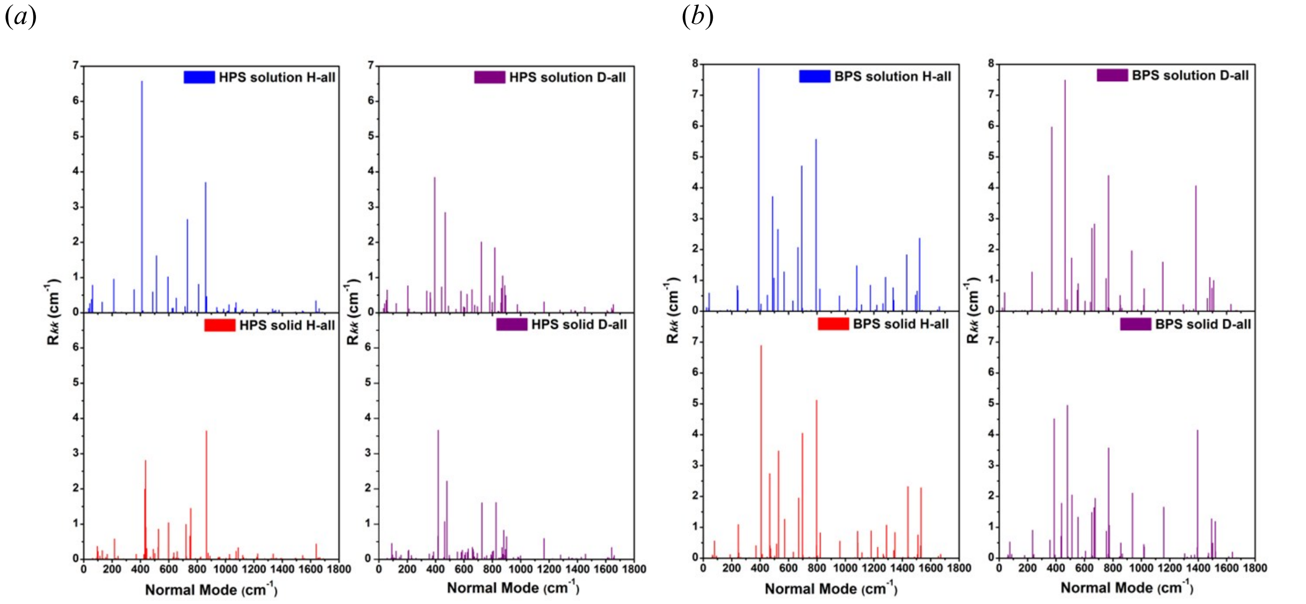

Fig. S1 Diagonal elements R_{kk} of the pre-factor R_{kl} versus the normal mode frequencies for non-deuterated (**H-all**) and fully-deuterated (**D-all**) isotopomers of **HPS** (*a*) and **BPS** (*b*) in both solution and solid phases.

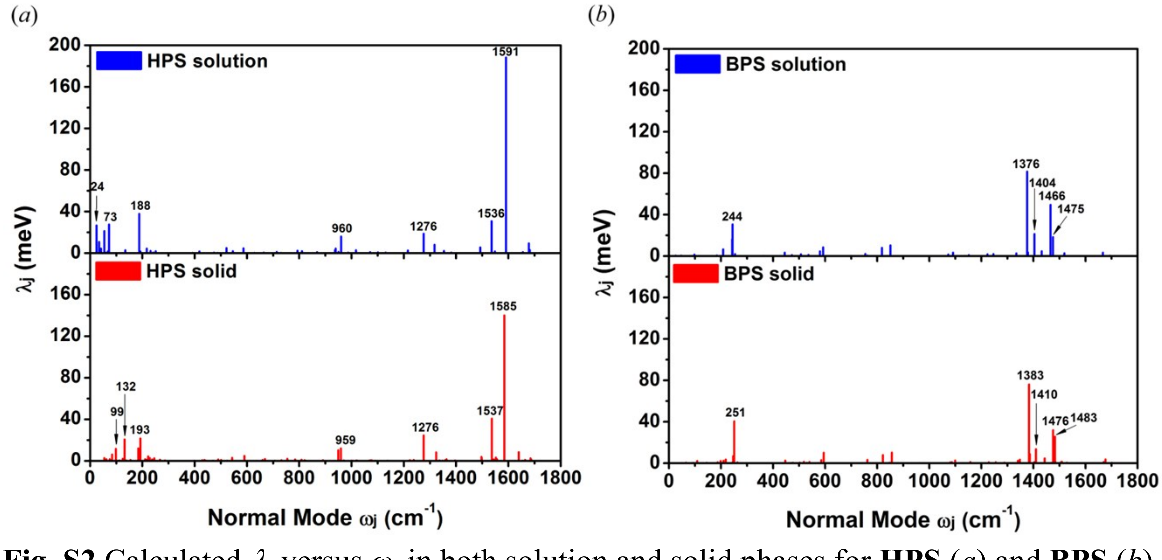

Fig. S2 Calculated λ_j versus ω_j in both solution and solid phases for **HPS** (*a*) and **BPS** (*b*).

Table S11 Selected normal modes j of $\mathbf{S_0}$ with large relaxation energies ($\lambda_j \geqq$ 1 meV), as well as frequencies of each mode ω_j, Huang-Rhys factors (S_j) for non-deuterated (**H-all**) and fully-deuterated (**D-all**) **HPS** in the solution phase.

**H-all**			**D-all**		
ω_j (cm^{-1})	S_j	λ_j (meV)	ω_j (cm^{-1})	S_j	λ_j (meV)
24	8.869	26.71	23	9.597	27.39
35	2.500	10.81	33	2.673	10.97
41	0.856	4.39	38	0.976	4.62
54	3.183	21.28	50	3.372	21.07
69	0.231	1.98	58	0.192	1.38
73	3.058	27.62	64	0.206	1.63
134	0.166	2.77	67	3.080	25.57
188	1.624	37.78	127	0.159	2.50
192	0.065	1.55	180	0.394	8.77
217	0.159	4.28	181	1.374	30.85
231	0.074	2.12	206	0.099	2.53
251	0.062	1.95	218	0.129	3.48
418	0.030	1.56	236	0.072	2.09
522	0.075	4.88	364	0.032	1.44
546	0.029	1.95	454	0.045	2.52
587	0.063	4.60	492	0.047	2.85
714	0.013	1.15	574	0.060	4.29
793	0.024	2.37	660	0.015	1.21
810	0.018	1.84	786	0.029	2.81
868	0.010	1.05	794	0.017	1.64
937	0.023	2.69	836	0.016	1.65
939	0.038	4.43	839	0.025	2.64
950	0.010	1.23	844	0.011	1.10
960	0.134	15.97	946	0.145	17.00
1017	0.022	2.77	977	0.016	1.95
1216	0.017	2.58	1224	0.112	16.97
1276	0.118	18.73	1277	0.061	9.64
1318	0.049	8.07	1388	0.016	2.69
1354	0.013	2.13	1426	0.051	9.06
1493	0.029	5.45	1444	0.024	4.30
1536	0.161	30.70	1583	1.050	206.05
1548	0.008	1.58	1612	0.013	2.52
1591	0.954	188.15	1619	0.021	4.20
1678	0.045	9.27	1644	0.061	12.53
1682	0.014	2.95	1650	0.034	6.89

Table S12 Selected normal modes j of $\mathbf{S_0}$ with large relaxation energies ($\lambda_j \geqq$ 1 meV), as well as frequencies of each mode ω_j, Huang-Rhys factors (S_j) for non-deuterated (**H-all**) and fully-deuterated (**D-all**) **HPS** in the solid phase.

**H-all**			**D-all**		
ω_j (cm^{-1})	S_j	λ_j (meV)	ω_j (cm^{-1})	S_j	λ_j (meV)
55	0.412	2.79	52	0.426	2.75
63	0.210	1.63	60	0.201	1.49
75	0.181	1.69	71	0.260	2.29
84	0.586	6.14	79	0.675	6.62
99	0.960	11.74	91	0.139	1.56
125	0.142	2.20	92	0.882	10.09
132	1.273	20.77	117	0.134	1.94
184	0.534	12.21	122	1.240	18.69
193	0.908	21.71	127	0.074	1.16
210	0.063	1.63	175	0.265	5.74
217	0.040	1.09	184	1.230	28.11
222	0.155	4.27	197	0.053	1.30
228	0.099	2.81	208	0.117	3.02
237	0.051	1.50	216	0.086	2.30
246	0.085	2.60	223	0.048	1.33
267	0.038	1.25	229	0.071	2.01
438	0.019	1.03	232	0.110	3.17
490	0.023	1.40	250	0.049	1.51
543	0.043	2.91	385	0.029	1.40
591	0.065	4.73	458	0.029	1.63
669	0.024	1.98	563	0.038	2.64
755	0.021	2.00	571	0.032	2.25
784	0.014	1.36	642	0.017	1.37
809	0.013	1.27	667	0.023	1.86
949	0.087	10.20	745	0.028	2.55
959	0.102	12.12	760	0.013	1.24
1239	0.007	1.07	845	0.012	1.27
1276	0.155	24.49	949	0.130	15.30
1324	0.050	8.25	1225	0.148	22.54
1363	0.009	1.56	1280	0.030	4.80
1497	0.021	3.82	1282	0.029	4.64
1498	0.016	2.97	1399	0.008	1.46
1537	0.214	40.78	1404	0.009	1.60
1541	0.007	1.32	1428	0.066	11.71
1544	0.007	1.43	1448	0.028	5.07
1552	0.017	3.27	1574	0.927	180.90
1556	0.008	1.56	1613	0.012	2.48
1585	0.713	140.13	1616	0.006	1.23

1639	0.041	8.40	1641	0.020	4.04
1684	0.013	2.65	1651	0.024	4.92
1688	0.007	1.43	1655	0.034	7.04

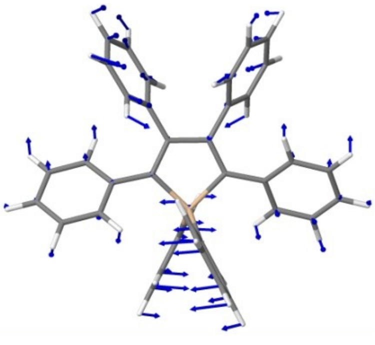

Mode 2, 24 cm^{-1}

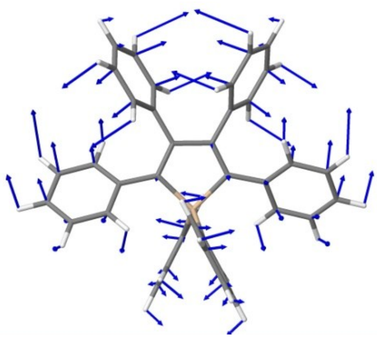

Mode 15, 73 cm^{-1}

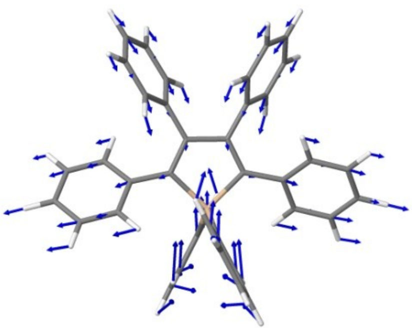

Mode 20, 188 cm^{-1}

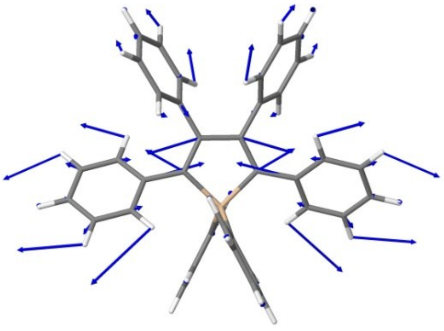

Mode 137, 1276 cm^{-1}

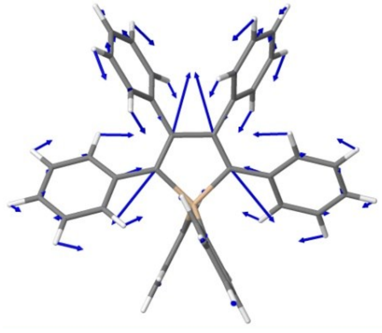

Mode 164, 1591 cm^{-1}

Fig. S3 Diagrammatic illustration of selected normal modes with large relaxation energies (λ_j) for $\mathbf{S_0}$ of solution-phase **HPS**.

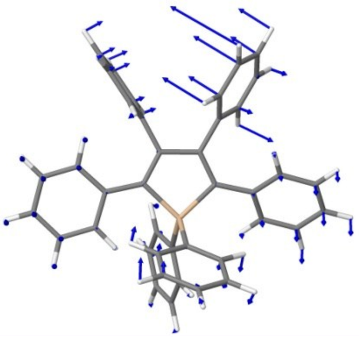

Mode 8, 99 cm^{-1}

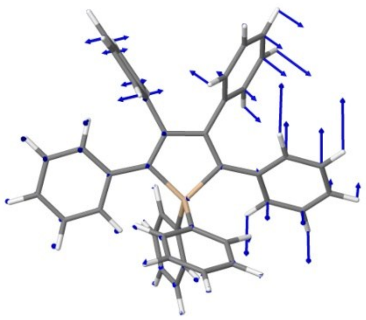

Mode 15, 132 cm^{-1}

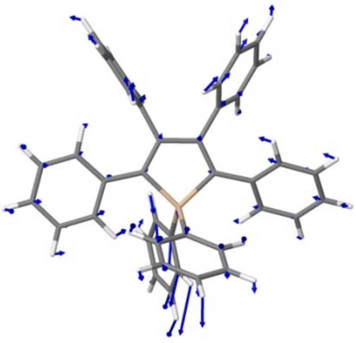

Mode 20, 193 cm^{-1}

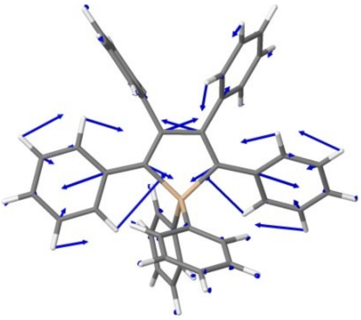

Mode 137, 1276 cm^{-1}

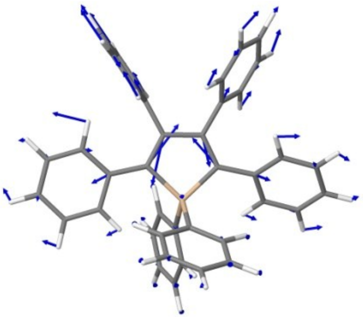

Mode 164, 1585 cm^{-1}

Fig. S4 Diagrammatic illustration of selected normal modes with large relaxation energies (λ_j) for $\mathbf{S_0}$ of solid-phase **HPS**.

Table S13 Selected normal modes *j* with large relaxation energies (λ_j), as well as frequencies of each mode ω_j, dimensionless displacement ($\Delta_j = \sqrt{\omega_j} D_j$) and Huang-Rhys factors (S_j) for ground-state **HPS** in both solution and solid phases. The corresponding vibration type is underlined.

mode *j*	ω_j (cm^{-1})	Δ_j	S_j	λ_j (meV)	Vibration types[a]
			**solution**		
2	24	4.212	8.869	26.71	i (2,5-rings twisting), ii (1,1,3,4-rings)
15	73	2.473	3.058	27.62	i (all benzene rings twisting)
20	188	-1.802	1.624	37.78	i (1,1-rings deformation), iii (CSiC, CCSi)
137	1276	-0.487	0.118	18.73	iv (2,5,3,4-rings), v (2,5-rings CC, silole CC and CSi)
164	1591	1.381	0.954	188.15	iv (2,5,3,4-rings), v (2,5,3,4-rings CC, silole CC), iii (CSiC)
			**solid**		
8	99	1.386	0.960	11.74	i (1,1,3,4-rings twisting), ii (2,5-ring)
15	132	1.596	1.273	20.77	i (2,5,3,4-rings twisting)
20	193	1.348	0.908	21.71	i (1,1-rings deformation), iii (CSiC, CCSi)
137	1276	0.556	0.155	24.49	iv (2,5,3,4-rings), v (2,5-rings CC, silole CC and CSi)
164	1585	-1.194	0.713	140.13	iv (2,5,3,4-rings), v (2,5,3,4-rings CC, silole CC), iii (CSiC)

[a]**Vibration types**: (**i**) benzene (heterocyclic) ring out-of-plane deformation/twisting vibration; (**ii**) CH out-of-plane rocking vibration; (**iii**) CCC (CSiC, CCSi) in-plane bending vibration; (**iv**) CH in-plane bending vibration; (**v**) CC (CSi) stretching vibration.

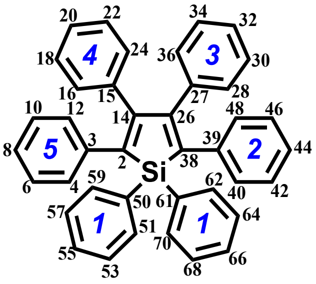

Table S14 Selected bond lengths (in Å), bond angles (in deg) and dihedral angles (in deg) of solution-phase and solid-phase **HPS** at the $\mathbf{S_0}$ ($\mathbf{S_1}$) optimized geometry.

	solution phase			solid phase			
	$\mathbf{S_0}$	$\mathbf{S_1}$	$\lvert\Delta(\mathbf{S_0}\text{-}\mathbf{S_1})\rvert$	$\mathbf{S_0}$	$\mathbf{S_1}$	$\lvert\Delta(\mathbf{S_0}\text{-}\mathbf{S_1})\rvert$	Crystal[a]
Si-C38	1.88	1.87	0.01	1.88	1.86	0.02	1.86
Si-C2	1.88	1.87	0.01	1.89	1.88	0.01	1.87
Si-C61	1.88	1.89	0.01	1.89	1.90	0.01	1.86
Si-C50	1.88	1.89	0.01	1.89	1.90	0.01	1.87
C38-C39	1.47	1.44	0.03	1.47	1.44	0.03	1.48
C2-C3	1.47	1.44	0.03	1.47	1.45	0.02	1.48
C26-C27	1.49	1.48	0.01	1.49	1.47	0.02	1.49
C14-C15	1.49	1.48	0.01	1.49	1.48	0.01	1.49
Si-C38-C26	107.18	108.80	1.62	106.72	108.60	1.88	107.14
C14-C26-C38	116.48	115.65	0.83	116.63	115.46	1.17	116.05
C2-Si-C38	92.61	91.06	1.55	93.14	91.39	1.75	93.22
C50-Si-C61	111.67	109.00	2.67	111.50	109.14	2.36	111.65
(**3**)C38-C26-C27-C28	57.91	53.34	4.57	61.09	53.50	7.59	58.54
(**4**)C2-C14-C15-C16	57.91	53.34	4.57	82.65	81.26	1.39	79.74
(**2**)Si-C38-C39-C40	43.95	28.41	15.54	42.97	37.73	5.24	43.96
(**5**)Si-C2-C3-C4	43.94	28.41	15.53	0.80	2.97	2.17	0.65
(**1**)C38-Si-C61-C62	-23.78	-22.02	1.76	-71.40	-70.61	0.79	-69.38
(**1**)C2-Si-C50-C59	-23.76	-22.01	1.75	30.56	28.98	1.58	27.86

[a]Ref. 8

Table S15 Selected normal modes j of $\mathbf{S_0}$ with large relaxation energies ($\lambda_j \geqq 1$ meV), as well as frequencies of each mode ω_j, Huang-Rhys factors (S_j) for non-deuterated (**H-all**) and fully-deuterated (**D-all**) **BPS** in the solution phase.

**H-all**			**D-all**		
ω_j (cm^{-1})	S_j	λ_j (meV)	ω_j (cm^{-1})	S_j	λ_j (meV)
98	0.108	1.31	93	0.123	1.42
208	0.251	6.47	201	0.277	6.89
243	0.541	16.31	225	0.047	1.31
244	1.011	30.62	234	0.089	2.59
254	0.062	1.95	236	1.437	42.12
445	0.059	3.27	417	0.065	3.33
507	0.028	1.75	431	0.045	2.42
536	0.018	1.19	486	0.019	1.17
580	0.064	4.58	519	0.019	1.21
592	0.114	8.40	563	0.069	4.82
754	0.022	2.05	581	0.104	7.47
819	0.078	7.93	726	0.020	1.79
851	0.098	10.28	751	0.107	9.98
1073	0.011	1.43	797	0.059	5.79
1091	0.024	3.31	898	0.015	1.64
1224	0.011	1.67	1041	0.011	1.46
1247	0.012	1.91	1314	0.277	45.11
1334	0.016	2.57	1346	0.006	1.04
1376	0.478	81.61	1383	0.047	8.11
1379	0.020	3.36	1398	0.300	51.94
1404	0.122	21.25	1457	0.358	64.73
1432	0.026	4.63	1465	0.090	16.37
1466	0.272	49.42	1503	0.008	1.43
1475	0.100	18.29	1510	0.021	3.86
1519	0.015	2.77	1636	0.015	3.04
1667	0.016	3.28			

Table S16 Selected normal modes *j* of $\mathbf{S_0}$ with large relaxation energies ($\lambda_j \geqq 1$ meV), as well as frequencies of each mode ω_j, Huang-Rhys factors (S_j) for non-deuterated (**H-all**) and fully-deuterated (**D-all**) **BPS** in the solid phase.

H-all			D-all		
ω_j (cm^{-1})	S_j	λ_j (meV)	ω_j (cm^{-1})	S_j	λ_j (meV)
109	0.163	2.19	103	0.184	2.37
198	0.090	2.21	188	0.070	1.64
210	0.090	2.34	198	0.058	1.43
218	0.129	3.49	208	0.226	5.83
247	0.223	6.82	228	0.039	1.11
251	1.301	40.46	239	0.451	13.36
447	0.044	2.46	243	0.950	28.67
518	0.021	1.33	246	0.056	1.71
539	0.019	1.25	419	0.062	3.23
585	0.041	2.94	435	0.025	1.33
595	0.138	10.16	492	0.022	1.35
762	0.034	3.21	522	0.019	1.20
822	0.076	7.79	567	0.076	5.33
856	0.096	10.19	583	0.037	2.68
1087	0.008	1.01	584	0.061	4.39
1099	0.019	2.64	732	0.026	2.38
1158	0.008	1.12	754	0.018	1.69
1340	0.015	2.43	755	0.089	8.31
1348	0.020	3.38	802	0.060	5.99
1383	0.444	76.07	904	0.014	1.62
1386	0.051	8.76	1044	0.009	1.18
1403	0.007	1.16	1320	0.263	43.01
1410	0.077	13.48	1322	0.012	1.89
1443	0.026	4.63	1353	0.010	1.68
1476	0.175	31.96	1394	0.048	8.35
1483	0.139	25.60	1396	0.013	2.27
1484	0.009	1.66	1404	0.010	1.71
1506	0.007	1.25	1407	0.162	28.34
1510	0.007	1.35	1407	0.101	17.64
1670	0.005	1.08	1469	0.262	47.68
1677	0.017	3.63	1473	0.084	15.30
			1474	0.041	7.57
			1494	0.014	2.58
			1640	0.005	1.08
			1646	0.016	3.30

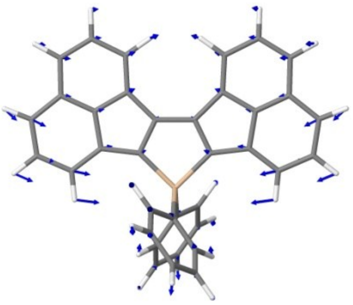
Mode 21, 244 cm^{-1}

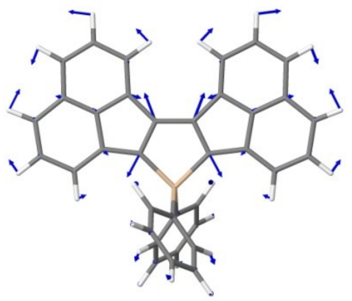
Mode 119, 1376 cm^{-1}

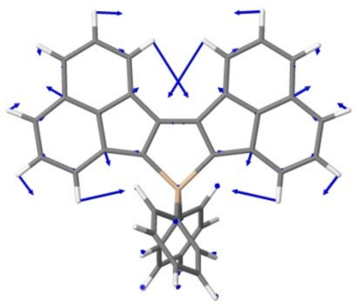
Mode 123, 1404 cm^{-1}

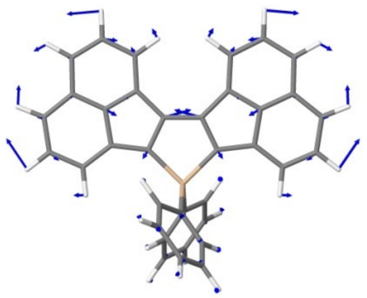
Mode 127, 1466 cm^{-1}

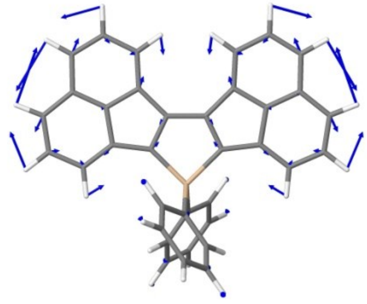
Mode 128, 1475 cm^{-1}

Fig. S5 Diagrammatic illustration of selected normal modes with large relaxation energies (λ_j) for $\mathbf{S_0}$ of solution-phase **BPS**.

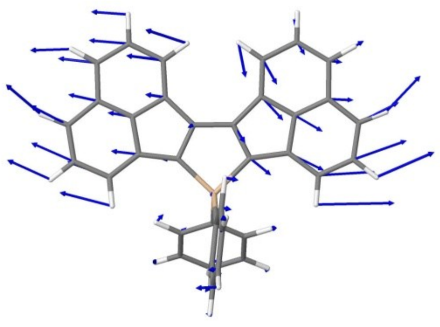
Mode 20, 251 cm^{-1}

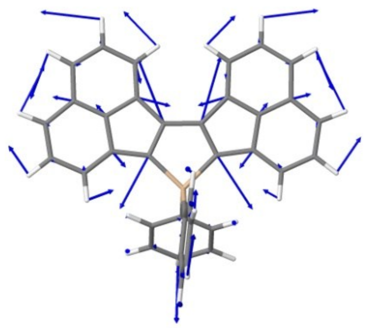
Mode 119, 1383 cm^{-1}

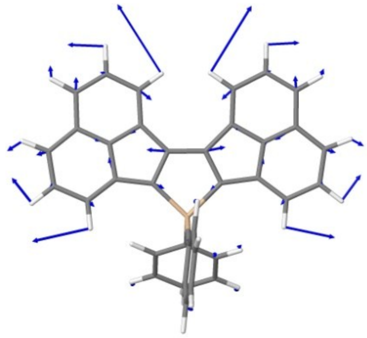
Mode 123, 1410 cm^{-1}

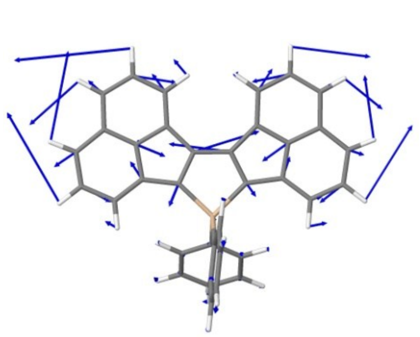
Mode 127, 1476 cm^{-1}

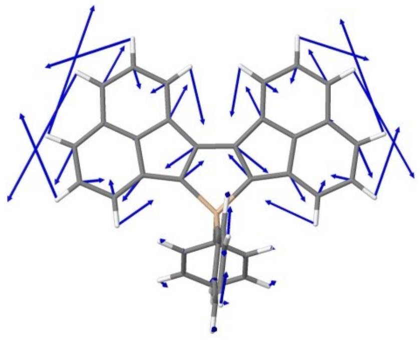
Mode 128, 1483 cm^{-1}

Fig. S6 Diagrammatic illustration of selected normal modes with large relaxation energies (λ_j) for $\mathbf{S_0}$ of solid-phase **BPS**.

Table S17 Selected normal modes j with large relaxation energies (λ_j), as well as frequencies of each mode ω_j, dimensionless displacement ($\Delta_j = \sqrt{\omega_j} D_j$) and Huang-Rhys factors (S_j) for ground-state **BPS** in both solution and solid phases. The corresponding vibration type is underlined.

**mode *j***	**ω_j (cm^{-1})**	**Δ_j**	**S_j**	**λ_j (meV)**	**Vibration types[a]**
			**solution**		
21	244	1.422	1.011	30.62	<u>i</u> (all benzene rings deformation), <u>iii</u> (CSiC)
119	1376	0.978	0.478	81.61	<u>iii</u> (2,3,4,5-rings CCC, CSiC), <u>iv</u> (all benzene rings), <u>v</u> (silole CC)
123	1404	0.494	0.122	21.25	<u>iii</u> (CCSi), <u>iv</u> (2,3,4,5-rings), <u>v</u> (2,3,4,5-rings CC and silole CC, CSi)
127	1466	0.737	0.272	49.42	<u>iii</u> (CSiC), <u>iv</u> (2,3,4,5-rings), <u>v</u> (2,3,4,5-rings CC and silole CC)
128	1475	-0.447	0.100	18.29	<u>iii</u> (CSiC), <u>iv</u> (2,3,4,5-rings), <u>v</u> (2,3,4,5-rings CC and silole CC)
			**solid**		
20	251	-1.613	1.301	40.46	<u>i</u> (all benzene rings deformation), <u>iii</u> (CSiC)
119	1383	0.942	0.444	76.07	<u>iii</u> (2,3,4,5-rings CCC, CSiC), <u>iv</u> (all benzene rings), <u>v</u> (silole CC)
123	1410	-0.393	0.077	13.48	<u>iii</u> (CCSi), <u>iv</u> (2,3,4,5-rings), <u>v</u> (2,3,4,5-rings CC and silole CC, CSi)
127	1476	0.591	0.175	31.96	<u>iii</u> (CSiC), <u>iv</u> (2,3,4,5-rings), <u>v</u> (2,3,4,5-rings CC and silole CC)
128	1483	-0.528	0.139	25.60	<u>iii</u> (CSiC), <u>iv</u> (2,3,4,5-rings), <u>v</u> (2,3,4,5-rings CC and silole CC)

[a]**Vibration types**: (**i**) benzene (heterocyclic) ring out-of-plane deformation/twisting vibration; (**ii**) CH out-of-plane rocking vibration; (**iii**) CCC (CSiC, CCSi) in-plane bending vibration; (**iv**) CH in-plane bending vibration; (**v**) CC (CSi) stretching vibration.

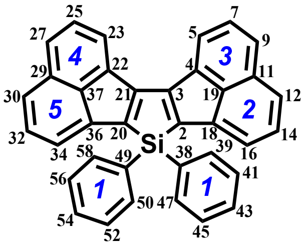

BPS

Table S18 Selected bond lengths (in Å), bond angles (in deg) and dihedral angles (in deg) of solution-phase and solid-phase **BPS** at the S_0 (S_1) optimized geometry.

	solution phase			solid phase			
	S_0	S_1	$\lvert\Delta(S_0\text{-}S_1)\rvert$	S_0	S_1	$\lvert\Delta(S_0\text{-}S_1)\rvert$	Crystal[a]
Si-C2	1.87	1.87	0.00	1.87	1.86	0.01	1.84
Si-C20	1.87	1.87	0.00	1.87	1.86	0.01	1.85
Si-C38	1.88	1.88	0.00	1.89	1.90	0.01	1.87
Si-C49	1.88	1.88	0.00	1.88	1.89	0.01	1.87
C2-C18	1.45	1.42	0.03	1.45	1.42	0.03	1.48
C20-C36	1.45	1.42	0.03	1.45	1.43	0.02	1.47
C3-C4	1.48	1.45	0.03	1.47	1.44	0.03	1.48
C21-C22	1.48	1.45	0.03	1.47	1.45	0.02	1.49
Si-C2-C3	109.14	110.94	1.80	108.65	110.90	2.25	109.32
C2-C3-C21	115.55	114.58	0.97	115.78	114.00	1.78	115.04
C2-Si-C20	90.59	88.96	1.63	91.26	89.41	1.85	91.34
C38-Si-C49	109.88	108.73	1.15	108.97	107.51	1.46	109.90
(3)C2-C3-C4-C19	0.56	0.33	0.23	0.94	0.94	0.00	0.58
(4)C20-C21-C22-C37	0.57	0.33	0.24	0.31	0.41	0.10	0.58
(2)Si-C2-C18-C16	1.97	1.29	0.68	0.92	0.87	0.05	3.86
(5)Si-C20-C36-C34	1.99	1.28	0.71	2.99	2.29	0.70	1.66
(1)C2-Si-C38-C39	-10.02	-12.84	2.82	45.09	44.43	0.66	41.78
(1)C20-Si-C49-C58	-10.41	-12.88	2.47	-35.37	-36.17	0.80	-40.16

[a]Ref. 13

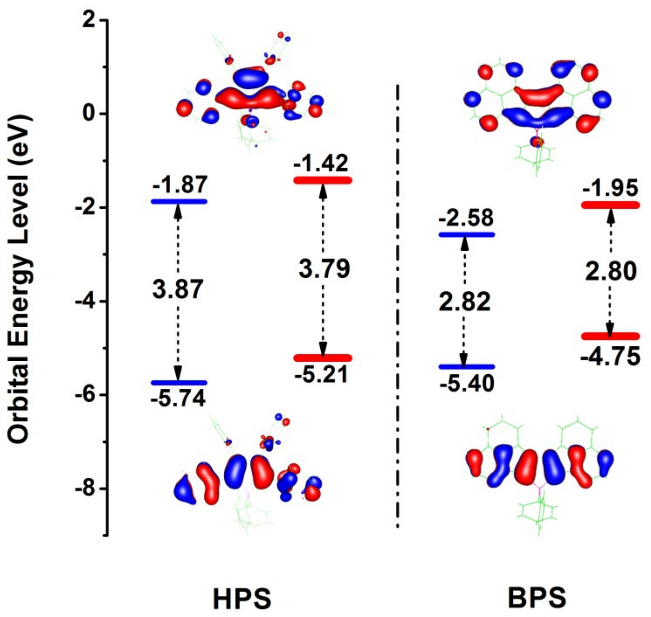

Fig. S7 HOMO and LUMO energy levels, HOMO-LUMO energy gaps, HOMO and LUMO contours for **HPS** and **BPS** at their $\mathbf{S_0}$ equlibrium geometries in both solution and solid phases.

Table S19 Room-temperature k_{ic} (s^{-1}) calculated without DRE for non-deuterated (**H-all**) and fully-deuterated (**D-all**) isotopomers of AIE-active **HPS** and AIE-inactive **BPS** in both solution and solid phases. The corresponding isotope effects (IE) are also given.

		HPS		BPS	
noDRE		solution	solid	solution	solid
k_{ic}	**H-all**	4.10×10^{6}	2.47×10^{4}	2.30×10^{8}	4.58×10^{8}
	**D-all**	4.76×10^{5}	7.06×10^{3}	1.20×10^{8}	2.38×10^{8}
IE		–88.4%	–71.4%	–47.8%	–48.0%

V. Synthesis of H- and D- HPS

THF was distilled from sodium benzophenone ketyl under dry nitrogen immediately prior to use. All chemicals and reagents were purchased from commercial sources and used as received without further purification. H- **HPS** was prepared according to published procedures.[8] D- **HPS** (Scheme S1): lithium granular (0.28 g, 40 mmol) was added dropwise into the dry THF solution of compound **1** (1.8 g, 10 mmol), and the resultant mixture was stirred for 6 h at room temperature, then the Ph_2SiCl_2 (6.04 g, 24 mmol) was added dropwise into the mixture. After reflux for 12 h, the mixture was poured into water and extracted with dichloromethane. The organic layer was washed successively with aqueous sodium chloride solution and water and then dried over magnesium

sulfate. After filtration, the solvent was evaporated underreduced pressure and the residue was purified by silica-gel column chromatography using hexane as eluent. Recrystallization gave a yellow solid of product in ~50% yield.

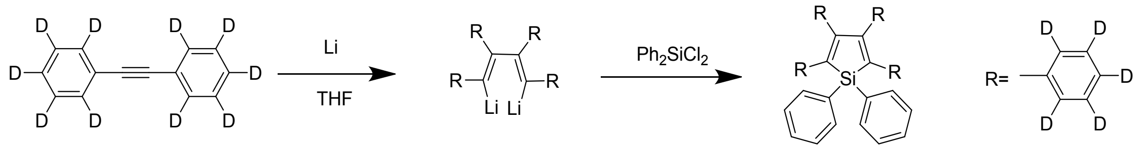

Scheme S1 Synthetic route of D- **HPS**.

^{1}H and ^{13}C NMR spectra were measured on a Bruker AV 500 spectrometer in deuterated chloroform with tetramethylsilane (TMS; δ=0) as internal reference. H- **HPS**: ^{1}H NMR (500 MHz, CD_2Cl_2),δ(TMS, ppm): 7.68-7.66 (m, 4H), 7.46-7.42 (m, 2H), 7.38-7.35 (m, 4H), 7.03–6.96 (m, 10H), 6.89-6.86 (m, 10H). ^{13}C NMR (125 MHz, CD_2Cl_2),δ(TMS, ppm): 156.7, 139.5, 138.7, 136.1, 131.6, 129.9, 129.2, 128.2, 127.7, 127.4, 126.4, 125.6. **D- HPS:** ^{1}H NMR (500 MHz, CD_2Cl_2),δ(TMS, ppm): 7.68 (d, J = 10.0 Hz, 10H), 7.60 (d, J = 10.0 Hz, 2H), 7.45-7.43 (m, 6H), 7.39–7.35 (m, 10H), 7.30-7.27 (m, 2H). ^{13}C NMR (125 MHz, CD_2Cl_2),δ(TMS, ppm): 156.7, 139.4, 139.3, 138.6, 136.1, 134.3, 131.6, 130.1, 128.2, 127.7.

VI. Computation for k_r and k_{ic} of H- and D- HPS

Theoretically, k_{ic} can be evaluated according to eqn S1 and k_r can be calculated through the simple Einstein spontaneous emission relationship of $k_r = \frac{fE_{vert}^2}{1.499\,s\cdot cm^{-2}}$,[S15] where f is the dimensionless oscillator strength of the excited state, E_{vert} is the vertical excitation energy from the excited state to the ground state with the dimension of cm^{-1}. A series of siloles have shown both considerably large energy gap (more than 1.4 eV) between $\mathbf{S_1}$ and $\mathbf{T_1}$ and very small spin-orbit coupling constants (less than 0.32 cm^{-1}) in our previous work,[26c] which indicates the rather slow

intersystem crossing process of **HPS** is negligible. Experimentally, k_r and k_{ic} can be derived from the measured values of the fluorescence quantum yield (Φ_F) and lifetime (τ_F). There exist $k_r = \Phi_F / \tau_F$ and $k_{ic} = (1 - \Phi_F)/\tau_F$. The measured Φ_F and τ_F before and after deuteration are listed in Table S20.

Table S20 Measured Φ_F and τ_F of **HPS** (298 K) before (H-) and after (D-) deuteration in both solution and solid phases.

	solution		solid	
**HPS**	Φ_F	τ_F	Φ_F	τ_F
H-	1.2%	0.94 ns	92.2%	6.07 ns
D-	1.5%	0.92 ns	96.7%	7.06 ns

VII. Partial Deuteration Effects for the 6-ring AIE analogues

Table S21 Room-temperature k_{ic} (s^{-1}) calculated for 1,1-rings or 1,6-rings only deuterated (**D-1,1**), 2,5-rings only deuterated (**D-2,5**), and 3,4-rings only deuterated (**D-3,4**) isotopomers of **HPS**, **BtTPS** and **HPDMCb** in both solution and solid phases, the corresponding isotope effect (IE) are given.

		**HPS**		**BtTPS**		**HPDMCb**	
	position	k_{ic}	IE	k_{ic}	IE	k_{ic} (s^{-1})	IE
**solution**	**D-1,1**	2.40×10^{11}	–1.6%	2.13×10^{11}	–3.2%	1.29×10^{11}	–1.5%
	**D-2,5**	2.34×10^{11}	–4.1%	2.08×10^{11}	–5.5%	1.30×10^{11}	–0.8%
	**D-3,4**	2.41×10^{11}	–1.2%	2.13×10^{11}	–3.2%	1.29×10^{11}	–1.5%
**solid**	**D-1,1**	8.02×10^{6}	–6.7%	2.70×10^{7}	–1.1%	1.54×10^{7}	–31.9%
	**D-2,5**	6.54×10^{6}	–24.0%	8.57×10^{6}	–68.6%	1.86×10^{7}	–17.7%
	**D-3,4**	4.16×10^{6}	–51.6%	2.53×10^{7}	–7.3%	1.56×10^{7}	–31.0%

Table S22 Effective frequencies (ω_{eff}) for 1,1-rings or 1,6-rings only deuterated (**D-1,1**), 2,5-rings only deuterated (**D-2,5**), and 3,4-rings only deuterated (**D-3,4**) isotopomers of **HPS**, **BtTPS** and **HPDMCb** in solid phases[a]

	ω_{eff}		
	**HPS**	**BtTPS**	**HPDMCb**
**D-1,1**	414 (–0.5%)	343 (–0.3%)	288 (–2.0%)
**D-2,5**	411 (–1.2%)	332 (–3.5%)	291 (–1.0%)
**D-3,4**	406 (–2.4%)	342 (–0.6%)	288 (–2.0%)

[a]The percentages of frequency reduction in the original frequencies are included in the parentheses.

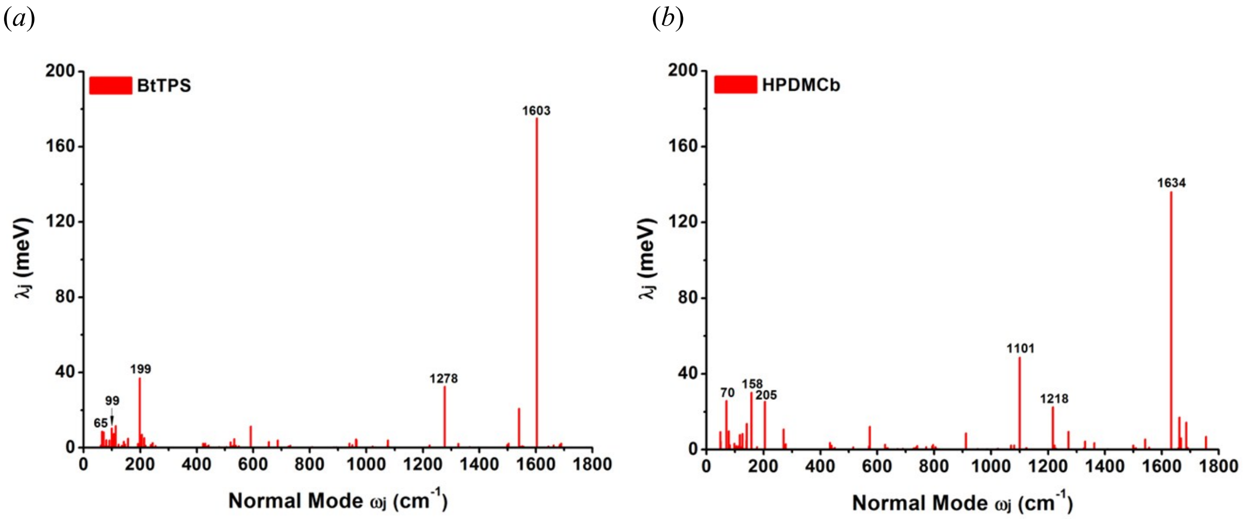

Fig. S8 Calculated λ_j versus ω_j in solid-phase **BtTPS** (*a*) and **HPDMCb** (*b*).

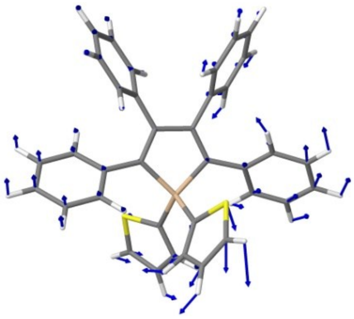

Mode 3, 65 cm^{-1}

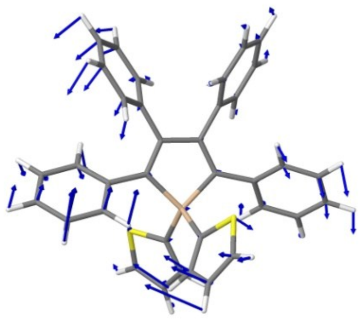

Mode 10, 99 cm^{-1}

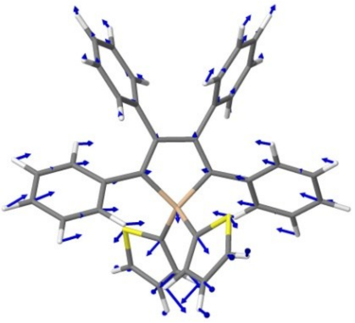

Mode 20, 199 cm^{-1}

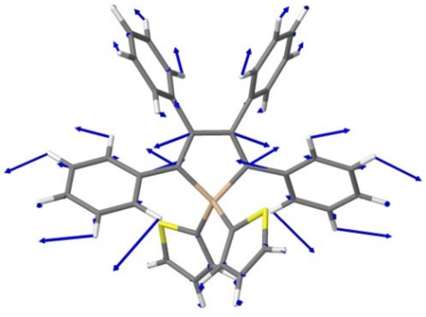

Mode 129, 1278 cm^{-1}

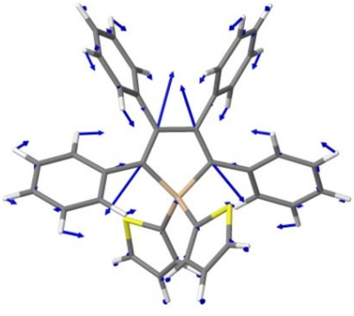

Mode 154, 1603 cm^{-1}

Fig. S9 Diagrammatic illustration of selected normal modes with large relaxation energies (λ_j) for $\mathbf{S_0}$ of solid-phase **BtTPS**.

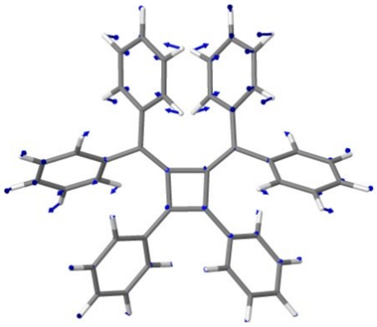

Mode 4, 70 cm^{-1}

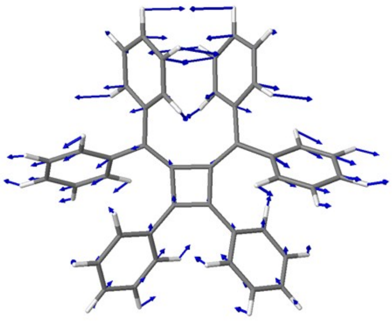

Mode 18, 158 cm^{-1}

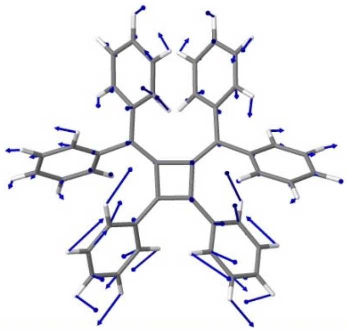

Mode 20, 205 cm^{-1}

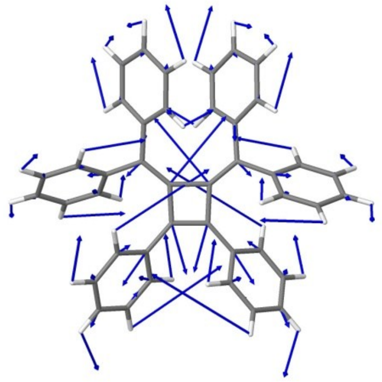

Mode 116, 1101 cm^{-1}

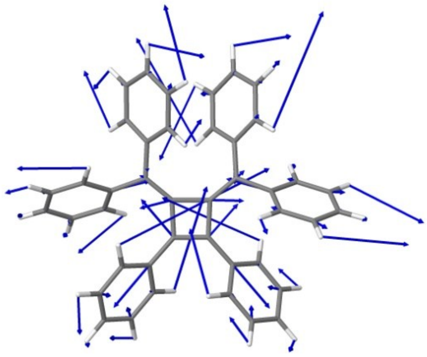

Mode 129, 1218 cm^{-1}

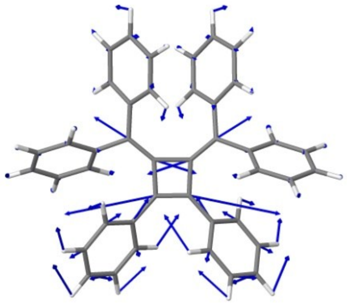

Mode 166, 1634 cm^{-1}

Fig. S10 Diagrammatic illustration of selected normal modes with large relaxation energies (λ_j) for $\mathbf{S_0}$ of solid-phase **HPDMCb**.

Table S23 Selected normal modes *j* with large relaxation energies (λ_j), as well as frequencies of each mode ω_j, dimensionless displacement ($\Delta_j = \sqrt{\omega_j} D_j$) and Huang-Rhys factors (S_j) for ground-state **BtTPS** and **HPDMCb** in solid phases. The corresponding vibration type is underlined.

mode *j*	ω_j (cm^{-1})	Δ_j	S_j	λ_j (meV)	Vibration types[a]
			**BtTPS**		
3	65	1.455	1.059	8.54	ii (all-rings)
10	99	-1.276	0.815	10.05	i (1,1,2,5-rings twisting), ii (3,4-ring)
20	199	1.764	1.556	38.37	i (1,1-rings deformation), iii (CSiC, CCSi)
129	1278	-0.565	0.159	25.27	iv (2,5-rings), iii (2,5-rings CCC, silole CCC and CSiC)
154	1603	1.332	0.887	176.39	iv (2,5,3,4-rings), v (2,5,3,4-rings CC, silole CC), iii (CSiC)
			**HPDMCb**		
4	70	-2.449	2.999	25.86	i (2,5,3,4-rings twisting)
18	158	1.756	1.541	30.12	i (3,4-rings deformation), iii (C38C2C3, C39C38C2)
20	205	-1.411	0.996	25.31	i (1,6,3,4-rings and cyclobutene deformation)
116	1101	-0.845	0.357	48.71	iv (all benzene rings), v (all benzene rings and cyclobutene), iii (cyclobutene)
129	1218	0.546	0.149	22.52	iv (all benzene rings), v (all benzene rings and cyclobutene)
166	1634	1.159	0.672	136.12	iv (1,6,3,4-rings), v (3,4-rings and cyclobutene), iii (cyclobutene)

[a]**Vibration types**: (**i**) benzene (heterocyclic) ring out-of-plane deformation/twisting vibration; (**ii**) CH out-of-plane rocking vibration; (**iii**) CCC (CSiC, CCSi) in-plane bending vibration; (**iv**) CH in-plane bending vibration; (**v**) CC (CSi) stretching vibration.

Table S24 Contributions from the internal coordinates of rings at the 1,1-positions, 2,5-positions and 3,4-positions with large λ_i ($\geqq$ 1 meV) to the total relaxation energy $\lambda_{g(e)}$ for **HPS** in solid phase.

**HPS**			
internal coordinates	$\lambda_{i(g)}$ (meV)	internal coordinates	$\lambda_{i(e)}$ (meV)
B(C39-C48)	3.03		
B(C39-C40)	2.68	D(C38-Si-C50-C59)	3.05
B(C3-C12)	2.07	D(C2-Si-C50-C51)	1.01
B(C3-C4)	2.60	**1,1-positions**	**4.06**
D(C26-C38-C39-C40)	4.39	B(C39-C48)	3.14
D(C1-C38-C39-C40)	4.59	B(C39-C40)	2.77
D(C26-C38-C39-C48)	3.52	B(C3-C12)	2.61
D(C1-C38-C39-C48)	3.61	B(C3-C4)	2.94
**2,5-positions**	**26.49**	D(C26-C38-C39-C40)	7.67
D(C38-C26-C27-C28)	6.14	D(C1-C38-C39-C40)	6.57
D(C38-C26-C27-C36)	5.67	D(C26-C38-C39-C48)	6.34
D(C14-C26-C27-C28)	3.91	D(C1-C38-C39-C48)	5.22
D(C14-C26-C27-C36)	3.54	**2,5-positions**	**37.26**
D(C26-C14-C15-C16)	2.64	D(C38-C26-C27-C28)	7.07
D(C26-C14-C15-C24)	2.82	D(C38-C26-C27-C36)	6.03
**3,4-positions**	**24.72**	D(C14-C26-C27-C28)	5.45
		D(C14-C26-C27-C36)	4.70
		D(C26-C14-C15-C16)	2.20
		D(C26-C14-C15-C24)	1.76
`		**3,4-positions**	**27.21**

Table S25 Contributions from the internal coordinates of rings at the 1,1-positions, 2,5-positions and 3,4-positions with large λ_i (≧ 1 meV) to the total relaxation energy $\lambda_{g(e)}$ for **BtTPS** in solid phase.

**BtTPS**			
internal coordinates	$\lambda_{i(g)}$ (meV)	internal coordinates	$\lambda_{i(e)}$ (meV)
D(C18-Si-C11-C10)	1.00	D(C18-Si-C11-C10)	4.40
D(C18-Si-C3-C4)	1.00	D(C18-Si-C3-C4)	3.95
D(C3-Si-C11-C10)	1.00	D(C3-Si-C11-C10)	1.52
**1,1-positions**	**3.00**	D(C54-Si-C3-S2)	2.04
B(C55-C56)	2.64	D(C54-Si-C11-C12)	1.02
B(C19-C28)	2.14	**1,1-positions**	**12.93**
B(C55-C64)	1.86	B(C55-C56)	2.47
B(C19-C20)	1.64	B(C19-C28)	2.13
D(Si-C18-C19-C28)	9.45	B(C55-C64)	2.05
D(Si-C18-C19-C20)	8.89	B(C19-C20)	1.75
D(Si-C54-C55-C56)	6.93	D(Si-C18-C19-C28)	16.90
D(Si-C54-C55-C64)	6.58	D(Si-C18-C19-C20)	16.53
D(C30-C18-C19-C28)	3.04	D(Si-C54-C55-C56)	10.41
D(C30-C18-C19-C20)	2.58	D(Si-C54-C55-C64)	9.13
D(C42-C54-C55-C56)	4.58	D(C30-C18-C19-C28)	10.81
D(C42-C54-C55-C64)	4.28	D(C30-C18-C19-C20)	10.50
**2,5-positions**	**54.61**	D(C42-C54-C55-C56)	8.34
D(C54-C42-C43-C52)	1.74	D(C42-C54-C55-C64)	7.13
D(C30-C42-C43-C52)	1.59	**2,5-positions**	**98.15**
D(C42-C30-C31-C32)	1.63	D(C54-C42-C43-C52)	2.47
D(C18-C30-C31-C32)	1.00	D(C30-C42-C43-C52)	2.48
D(C30-C42-C43-C44)	1.00	D(C42-C30-C31-C32)	2.29
D(C54-C42-C43-C44)	1.00	D(C18-C30-C31-C32)	1.53
**3,4-positions**	**7.96**	D(C30-C42-C43-C44)	1.56
		D(C54-C42-C43-C44)	1.47
		**3,4-positions**	**11.80**

Table S26 Contributions from the internal coordinates of rings at the 1,6-positions, 2,5-positions and 3,4-positions with large λ_i (≧ 1 meV) to the total relaxation energy $\lambda_{g(e)}$ for **HPDMCb** in solid phase.

**HPDMCb**			
internal coordinates	$\lambda_{i(g)}$ (meV)	internal coordinates	$\lambda_{i(e)}$ (meV)
B(C4-C13)	1.62	B(C4-C13)	1.69
B(C40-C49)	1.53	B(C40-C49)	1.66
B(C4- C5)	1.02	B(C4-C5)	1.20
B(C40-C41)	1.00	B(C40-C41)	1.20
D(C1-C37-C40-C49)	9.12	A(C1-C4-C5)	2.15
D(C37-C1-C4-C13)	10.31	A(C37-C40-C41)	2.09
D(C37-C1-C4-C5)	11.67	D(C1-C37-C40-C49)	23.26
D(C1-C37-C40-C41)	8.93	D(C37-C1-C4-C13)	21.84
D(C38-C37-C40-C49)	4.57	D(C37-C1-C4-C5)	17.26
D(C2-C1-C4-C13)	4.67	D(C1-C37-C40-C41)	18.55
D(C2-C1-C4-C5)	5.58	D(C38-C37-C40-C49)	10.41
D(C38-C37-C40-C41)	4.58	D(C2-C1-C4-C13)	9.60
**1,6-positions**	**64.60**	D(C2-C1-C4-C5)	7.04
D(C38-C39-C51-C52)	2.06	D(C38-C37-C40-C41)	7.81
D(C2-C3-C15-C16)	2.18	**1,6-positions**	**125.76**
D(C38-C39-C51-C60)	1.70	D(C38-C39-C51-C52)	2.57
D(C2-C3-C15-C24)	1.60	D(C2-C3-C15-C16)	2.45
**2,5-positions**	**7.54**	D(C38-C39-C51-C60)	1.75
B(C26-C35)	3.03	D(C2-C3-C15-C24)	1.73
B(C62-C71)	3.06	**2,5-positions**	**8.50**
B(C62-C63)	2.43	B(C26-C35)	2.47
B(C26-C27)	2.38	B(C62-C71)	2.39
B(C65-C67)	1.36	B(C62-C63)	1.92
B(C29-C31)	1.29	B(C26-C27)	1.83
D(C51-C39-C62-C71)	14.81	D(C51-C39-C62-C71)	14.36
D(C15-C3-C26-C35)	15.06	D(C15-C3-C26-C35)	13.63
D(C38-C39-C62-C71)	8.46	D(C38-C39-C62-C71)	8.90
D(C2-C3-C26-C35)	9.01	D(C2-C3-C26-C35)	7.93
D(C51-C39-C62-C63)	6.44	D(C51-C39-C62-C63)	7.84
D(C15-C3-C26-C27)	6.58	D(C15-C3-C26-C27)	7.50
D(C38-C39-C62-C63)	2.05	D(C38-C39-C62-C63)	3.90
D(C2-C3-C26-C27)	2.41	D(C2-C3-C26-C27)	3.38
**3,4-positions**	**78.37**	**3,4-positions**	**76.05**

VIII. Excitonic Couplings for HPS and BPS

We calculate the Coulomb coupling component of the excitonic coupling[S16] at the TD-CAMB3LYP[S17] level for **HPS** and **BPS** in the MOMAP[25] program based on the electronic structure information obtained from NWChem 6.3[S18].

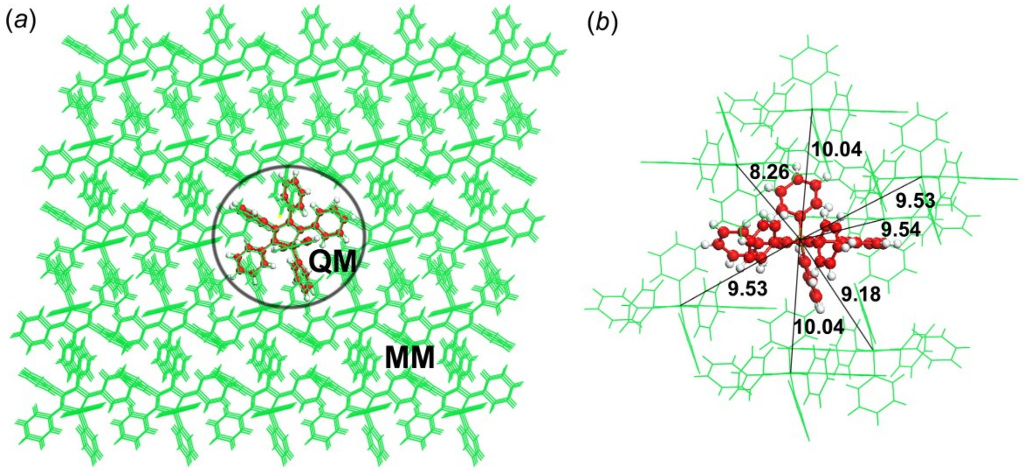

Fig. S11 (a) Setup of the QM/MM model for **HPS**. (b) Close look at the packing structures and the intermolecular distances (Å) within 10.10 Å of the QM centroid.

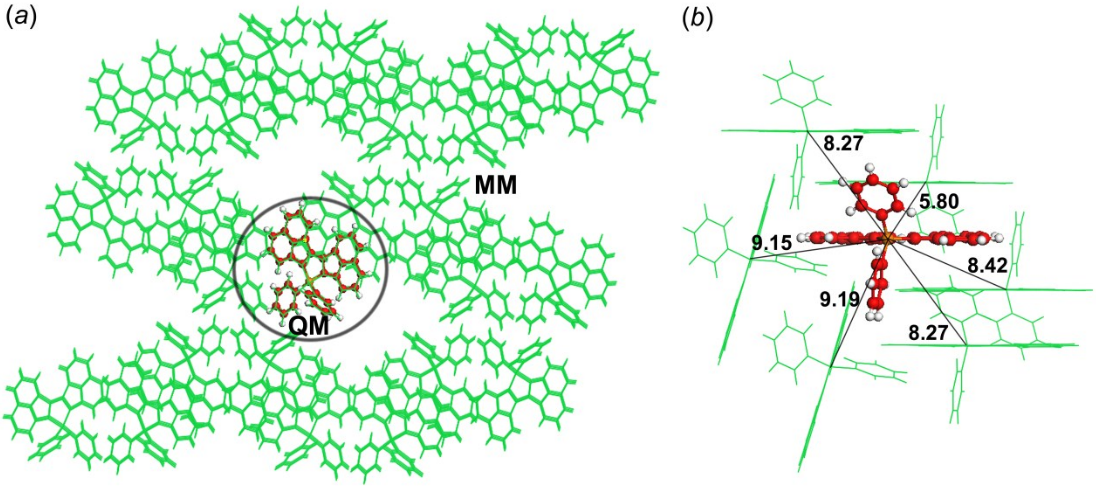

Fig. S12 (a) Setup of the QM/MM model for **BPS**. (b) Close look at the packing structures and the intermolecular distances (Å) within 10.00 Å of the QM centroid.

Table S27 Intermolecular distance (Fig. S11-S12) versus the Coulomb coupling component of the excitonic coupling (J_{coul}) in **HPS** and **BPS** cluster.

**HPS**		**BPS**	
Distance (Å)	J_{coul} (meV)	Distance (Å)	J_{coul} (meV)
8.26	7.09	5.80	19.02
9.18	14.84	8.27	4.11
9.53	11.33	8.42	4.66
9.54	12.06	9.15	7.53
10.04	8.30	9.19	7.36

Table S28 $\lambda_{g(e)}$ obtained by normal mode analysis for **HPS** and **BPS** in both solution and solid phases at the PBE0/6-31G* level.

	**HPS**		**BPS**	
	solution	solid	solution	solid
λ_g (meV)	469	407	299	293
λ_e (meV)	517	411	292	277

References

S1 M. J. Frisch, G. W. Trucks, H. B. Schlegel, G. E. Scuseria, M. A. Robb, J. R. Cheeseman, G. Scalmani, V. Barone, B. Mennucci, G. A. Petersson, H. Nakatsuji, M. Caricato, X. Li, H. P. Hratchian, A. F. Izmaylov, J. Bloino, G. Zheng, J. L. Sonnenberg, M. Hada, M. Ehara, K. Toyota, R. Fukuda, J. Hasegawa, M. Ishida, T. Nakajima, Y. Honda, O. Kitao, H. Nakai, T. Vreven, J. A. Montgomery, Jr., J. E. Peralta, F. Ogliaro, M. Bearpark, J. J. Heyd, E. Brothers, K. N. Kudin, V. N. Staroverov, R. Kobayashi, J. Normand, K. Raghavachari, A. Rendell, J. C. Burant, S. S. Iyengar, J. Tomasi, M. Cossi, N. Rega, J. M. Millam, M. Klene, J. E. Knox, J. B. Cross, V. Bakken, C. Adamo, J. Jaramillo, R. Gomperts, R. E. Stratmann, O. Yazyev, A. J. Austin, R. Cammi, C. Pomelli, J. W. Ochterski, R. L. Martin, K. Morokuma, V. G. Zakrzewski, G. A. Voth, P. Salvador, J. J. Dannenberg, S. Dapprich, A. D. Daniels, Ö. Farkas, J. B. Foresman, J. V. Ortiz, J. Cioslowski and D. J. Fox, *Gaussian 09*, *Revision D.01*, Gaussian, Inc., Wallingford CT, 2009.

S2 (*a*) P. D. Nguyen, F. Ding, S. A. Fischer, W. Liang and X. Li, *J. Phys. Chem. Lett.* 2012, **3**, 2898; (*b*) F. Ding, B. E. Van Kuiken, B. E. Eichinger and X. Li, *J. Chem. Phys.* 2013, **138**, 064104.

S3 A. Petrone, G. Donati, P. Caruso and N. Rega, *J. Am. Chem. Soc.* 2014, **136**, 14866.

S4 P. Sherwood, A. H. de Vries, M. F. Guest, G. Schreckenbach, C. R. A. Catlow, S. A. French, A. A. Sokol, S. T. Bromley, W. Thiel, A. J. Turner, S. Billeter, F. Terstegen, S. Thiel, J. Kendrick, S. C. Rogers, J. Casci, M. Watson, F. King, E. Karlsen, M. Sjøvoll, A. Fahmi, A. Schäfer and C. Lennartz, *J. Mol. Struct. :Theochem* 2003, **632**, 1.

S5 R. Ahlrichs, M. Bär, M. Häser, H. Horn and C. Kölmel, *Chem. Phys. Lett.* 1989, **162**, 165.

S6 D. Bakowies and W. Thiel, *J. Phys. Chem.* 1996, **100**, 10580.

S7 W. Smith and T. R. Forester, *J. Mol. Graph.* 1996, **14**, 136.

S8 J. M. Wang, R. M. Wolf, J. W. Caldwell, P. A. Kollman and D. A. Case, *J. Comput. Chem.* 2004, **25**, 1157.

S9 J. W. Ochterski, *Vibrational Analysis in Gaussian*; Gaussian, Inc.: Wallingford, CT, 1999.

S10 I. L. Karle and K. S. Dragonette, *Acta Crystallogr.* 1965, **19**, 500.

S11 C. Nather, H. Bock, Z. Havlas and T. Hauck, *Organometallics* 1998, **17**, 4707.

S12 R. Mason, *Acta Crystallogr.* 1964, **17**, 547.

S13 C. Adamo and D. Jacquemin, *Chem. Soc. Rev.* 2013, **42**, 845.

S14 R. Send and F. Furche, *J. Chem. Phys.* 2010, **132**, 044107.

S15 (*a*) E. Wolf, *Progress in Optics*; North-Holland Publishing Company, AmsterDam-London, 1972; (*b*) N. J. Turro, V. Ramamurthy and J. C. Scaiano, *Modern Molecular Photochemistry of Organic Molecules*, University Science Books: Sausalito, CA, 2010.
S16 C.-P. Hsu, Z.-Q. You and H.-C. Chen, *J. Phys. Chem. C* 2008, **112**, 1204.
S17 T. Yanai, D. P. Tew and N. C. Handy, *Chem. Phys. Lett.* 2004, **393**, 51.
S18 M. Valiev, E. J. Bylaska, N. Govind, K. Kowalski, T. P. Straatsma, H. J. J. Van Dam, D. Wang, J. Nieplocha, E. Apra, T. L. Windus and W. A. de Jong, *Comput. Phys. Commun.* 2010, **181**, 1477.
